# Integrated diagnostic network construction reveals a 4-gene panel and 5 cancer hallmarks driving breast cancer heterogeneity

**DOI:** 10.1038/s41598-017-07189-6

**Published:** 2017-07-28

**Authors:** Xiaofeng Dai, Tongyan Hua, Tingting Hong

**Affiliations:** 1 National Engineering Laboratory for Cereal Fermentation Technology, Jiangnan University, Wuxi, China; 2 The Key Laboratory of Industrial Biotechnology, Ministry of Education, School of Biotechnology, Jiangnan University, Wuxi, China; 3 Department of medical oncology, the affiliated hospital of Jiangnan University, the fourth people’s hospital of Wuxi, Wuxi, China

## Abstract

Breast cancer encompasses a group of heterogeneous diseases, each associated with distinct clinical implications. Dozens of molecular biomarkers capable of categorizing tumors into clinically relevant subgroups have been proposed which, though considerably contribute in precision medicine, complicate our understandings toward breast cancer subtyping and its clinical translation. To decipher the networking of markers with diagnostic roles on breast carcinomas, we constructed the diagnostic networks by incorporating 6 publically available gene expression datasets with protein interaction data retrieved from BioGRID on previously identified 1015 genes with breast cancer subtyping roles. The Greedy algorithm and mutual information were used to construct the integrated diagnostic network, resulting in 37 genes enclosing 43 interactions. Four genes, *FAM134B*, *KIF2C*, *ALCAM*, *KIF1A*, were identified having comparable subtyping efficacies with the initial 1015 genes evaluated by hierarchical clustering and cross validations that deploy support vector machine and *k* nearest neighbor algorithms. Pathway, Gene Ontology, and proliferation marker enrichment analyses collectively suggest 5 primary cancer hallmarks driving breast cancer differentiation, with those contributing to uncontrolled proliferation being the most prominent. Our results propose a 37-gene integrated diagnostic network implicating 5 cancer hallmarks that drives breast cancer heterogeneity and, in particular, a 4-gene panel with clinical diagnostic translation potential.

## Introduction

Despite the considerable contributions of traditional diagnostic and treatment modalities made in the battle against breast cancer, it still remains as the leading cause of women death worldwide^1, 2^. Though, if diagnosed early and treated appropriately, breast cancer patients have relatively better outcomes than many other types of malignancies, it is difficult to reach accurate diagnosis and optimal therapeutic design given distinct patients’ morphological features and treatment responses^3–7^. Canonically, breast carcinomas are grouped as luminal (luminal A and B), HER2 positive, and triple negative subtypes based on the status of estrogen receptor (ER), progesterone receptor (PR) and epidermal growth factor receptor 2 (HER2). While luminal tumors respond well to the hormonal therapy Tamoxifen^8^, and HER2 positive cancers could be properly treated with Herceptin^9^, triple negative breast cancers do not actively react to any available targeted modalities without severe adverse effects due to, primarily, lack of the three primary surface receptors^10–13^.

The diverse clinical consequences of breast cancer patients have led to a surge in the exploration of novel biomarkers and subtyping strategies of this complicated disease^7^. For example, androgen receptor (AR) was used, instead of HER2, to classify ER-PR- breast cancers into ER−PR−AR+, ER−PR−AR− subclasses with distinct clinical features^14^. The additional use of proliferation markers KI67^15^ and/or TOP2A^16^ with the conventional diagnostic modality has led to improved accuracy of identifying luminal A from B tumors. Lots of efforts have been devoted to sub-classify the triple negative group (TNG). While some studies use cytokeratins such as CK5/6^17–23^, CK14^22^, CK17^22^, CK8/18^19^ to differentiate the basal subtype from the rest TNG tumors, some find EGFR^17–20, 22^, vimentin^19^, P-cadherin^21^ or TP63^21^ effective for this purpose. With the diverse biomarkers identified, the number of breast cancer subtypes varies considerably among studies^24^. These though contribute in deciphering breast cancer heterogeneity, considerably complicate our understandings toward breast cancer differentiation and hamper their clinical translations.

Biomarkers identified from networks are reported more reproducible than individual ones selected without network information^25^. An integrated network has been considered useful to integrate multiple levels of high-throughput information and gain comprehensive understandings of cancer related genomic alterations^26^. Tumor clonal network, by treating tumor as an evolving system and computationally dissecting clones from tumors, has been proposed as an effective tool to gain a ‘whole-system’ view of a tumor for personalized cancer management^27, 28^. Ever since 2011 when Weinberg brought up the concept of cancer hallmarks, targeting the hallmarks of cancer has been considered as a rational approach to the next-generation cancer therapy^29^. Accordingly, cancer hallmark network has opened a novel window for predicting patient clinical outcome from a myriad of phenotypic complexities governed by a limited set of organizing principles^30^. Under this framework, a set of mutations and copy number variations were reported effective in predicting subtype-specific drug targets in breast cancer^31^; and cancer hallmark-based gene signature sets were identified beneficial in predicting the recurrence and chemotherapy response of stage II colorectal cancer patients^32^.

Inspired by these previous efforts we, in this paper, focus on identifying genes and hallmarks governing the heterogeneity of breast cancer from the network point of view. For this, we constructed six diagnostic networks by integrating each of 6 publically available gene expression datasets with protein interaction data retrieved from BioGRID^33^ on 1015 diff-genes previously reported with breast cancer subtyping roles^34^. Using the Greedy algorithm and mutual information we condensed each of the 6 networks, and merged genes present in at least three networks to preserve as much information as possible with the most succinct number of genes.

The resulting integrated diagnostic network contains 37 genes and 43 interactions, among which four, i.e., *FAM134B*, *KIF2C*, *ALCAM*, *KIF1A*, were identified with comparable subtyping efficacies with the initial 1015 genes (which were evaluated by hierarchical clustering and leave-one-out cross validations). Pathway, Gene Ontology, and proliferation marker enrichment analyses reveal five critical cancer hallmarks driving the complexity and heterogeneity of breast cancers, which are ‘enabling replicative immortality’, ‘sustaining proliferative signaling’, ‘resisting cell death’, ‘deregulating cellular energetics’, and ‘activating invasion & metastasis’. Our results offer a 4-gene panel with feasible size for clinical translation, and underpin 5 cancer hallmarks and associated pathways for therapeutic design. These not only update our knowledge toward breast cancer complexity and, more importantly, provide practical insights and tools for breast cancer control.

## Methods

### Construction of the diff-gene protein network

Protein interactions (PPI) of 1015 genes differentiating breast cancer subtypes (diff-genes) proposed in ref. 34 were retrieved from the public database BioGRID (Biology General Repository for Interaction Datasets)^33^ and used for ‘diff-gene protein network’ construction. BioGRID version 3.4.147 was requested which encompasses 1,421,025 protein and genetic interactions, 27,785 chemical associations and 38,559 post-translational modifications of major modelling organisms from 58,514 papers.

### Construction of diagnostic diff-gene networks

Six datasets, GSE70947, GSE15852, GSE20711, GSE65212, GSE18229-GPL887, GSE65194, were retrieved from the GEO database (Gene Expression Omnibus)^35^ and included in this study. We conducted the analysis using data free of metastasis. Two datasets are comprised of case-control sample pairs (i.e., each pair is consist of one breast cancer tissue sample and its adjacent normal breast tissue), with GSE70947 and GSE15852 each encompassing 148 and 43 sample pairs. GSE20711 contains 88 breast cancer and 2 normal breast tissue samples. We removed cell line and mammoplasty data from GSE65212, GSE18229-GPL887 and GSE65194, and kept 164 (out of 178) samples from GSE65212 (comprised of 153 breast cancer and 11 normal breast tissue samples), 77 (out of 94) samples from GSE18229-GPL887 (including 72 breast cancer and 5 normal tissue samples), and 165 (out of 178) samples from GSE65194 (composed of 153 breast cancer and 12 normal breast tissue samples) for diff-gene network construction.

Differential expression analysis of the diff-genes between breast cancer and normal samples was conducted for each dataset using GEO2R, which is an interactive web tool allowing comparisons between two or among multiple groups of samples in a GEO series using limma R packages on the original submitter-supplied processed data^36–38^. Diff-genes differentially expressed in breast cancer tissues obtained using each dataset are believed to capture subtyping features and specific to tumor cells. They are considered with more profound diagnostic values and named ‘diagnostic diff-genes’ here.

The p-values of these diagnostic diff-genes were corrected using the Benjamini & Hochberg adjustment method and transformed to paired t-scores using Equation 1,

1 $${t}_{g}=\frac{{\bar{X}}_{g1}-{\bar{X}}_{g2}}{\sqrt{\frac{{{S}_{g1}}^{2}}{{n}_{1}}+\frac{{{S}_{g2}}^{2}}{{n}_{2}}}}\,,$$

where ${\bar{X}}_{gi}=\sum _{j=1}^{{n}_{i}}\frac{{X}_{gij}}{{n}_{i}}\,,$ ${{S}_{gi}}^{2}=\frac{1}{{n}_{i}-1}\sum _{j=1}^{{n}_{i}}{({X}_{gij}-{\bar{X}}_{gi})}^{2},\,{X}_{gij}$ denotes the expression level of *g* ^th^ gene in the *i* ^th^ sample and j^th^ experiment, and *n*_*i*_ represents the sample size of each sample cohort. The higher the t-score of a given gene is the more significant diagnostic value the gene is associated with.

### Construction of diagnostic networks

Each diagnostic diff-gene network was combined with the diff-gene protein network by keeping edges in common, forming six independent diagnostic networks. The Greedy searching strategy based on mutual information was employed to find the most succinct network maintaining the highest accumulated t-score for each diagnostic network using the jActiveModules plugin in Cytoscape^39^. Mutual information is computed by Equation 2, where *a* and *c* each denotes the nodes, *x* and *y* each represents the t-scores of *a* and *c*, *p*(*x*, *y*) is the probability density function of *a* and *c*, *p*(*x*) and *p*(*y*) are the partial probability density function of *a* and *c*, respectively.

2 $${\rm{S}}(M)=MI(a^{\prime} ,c)=\sum _{x\in a}\sum _{y\in c}p(x,y)\mathrm{log}\,\frac{p(x,y)}{p(x)p(y)}$$

The Greedy algorithm is an iterative approach where, in each round, it randomly selects one node (seed), expands the network by adding nodes that raise the overall t-score until no further increase is obtainable. The top 10 sub-networks (ranked by t-scores) were merged after generating ‘*n*_0_’ (the number of genes in the initial network) sub-networks with each gene as the seed, resulting in a network containing ‘*n*_*r*_’ genes (*r* denotes the *r* ^th^ run). Multiple rounds of the Greedy algorithm were run using ‘*n*_*r*−1_’ nodes as the starting network until *n*_*r*_ meets the stopping criterion which was set to approximately 50 here.

### Construction of integrated diagnostic network

The overlapping rate was computed for each combination of the six diagnostic networks using Equation 3

3 $${\rm{Overlapping}}\,{\rm{rate}}=\frac{{G}^{1\ldots n}}{{G}^{1}+{G}^{2}+\cdots {G}^{n}-{G}^{1\ldots n}}\times 100 \% ,$$

where *n* ranges from 2 to 6, *G* ^1^, *G* ^2^ and *G* ^*n*^ each denotes the number of genes in the *n* ^th^ diagnostic network under comparison, and *G* ^1…*n*^ denotes the genes in common among the *n* compared networks. Genes and edges present in at least three diagnostic networks were selected as the integrated diagnostic network.

### Identification and evaluation of pivotal diagnostic genes

#### Connectivity assessment

The degee of each node, i.e., the number of edges each gene connects with its neighbors, was asssessed to measure the importance of each identified diagnostic diff-gene. Genes were categorized into <25%, 25–50%, 50–75%, >75% quantiles of the degree distribution, i.e., genes with 1–12, 13–35, 36–76 or >76 degrees were grouped into distinct classes. BioGRID contains 13369 nodes and 109670 edges after the removal of singletons, with the node degree ranges from 1 to 3576. We computed the percentage of each group of identified diagnostic diff-genes represented in BioGRID (*Per*_*i*_) using Equation 4

4 $$Pe{r}_{i}=\frac{{{\rm{N}}}_{s,i}}{{{\rm{N}}}_{D,i}},$$

where *N*_*s,i*_ represents the number of diagnostic diff-genes in level *i* and *N*_*D,i*_ represents the number of genes in BioGRID fell in level *i*. Permutation test with 1000 runs was conducted to evaluate whether genes in the highly connected group (>75% percentile) are obtained by chance.

The enrichment of the connecitivity for gene *j* (*EC*_*j*_) from the integrated diagnostic network was computed using $E{C}_{j}=\frac{{C}_{s,j}}{{{\rm{C}}}_{D,j}}$ (*C*_*s*,*j*_ represents the number of connectivity of gene *j* in the integrated diagnostic network, and C_*D,j*_ represents the connectivity of gene *j* in BioGRID).

Genes whose connectivity is highly enriched in the integrated diagnostic network were considered specific to and crucial for breast cancer diagnosis, and were selected as candidate ‘pivotal diagnostic genes’.

#### Patient survival association study

Kaplan Meier Plotter^40^ (http://kmplot.com/analysis/index.php?p=service&cancer=breast), a database containing clinical information and gene expression data on 3951 breast cancer patients, was used to evaluate the clinical association of each candidate pivotal diagnostic gene with breast cancer patient 10-year relapse free survival. Genes without significant association with patient survival were excluded from the pivotal diagnostic gene panel.

#### Cross validation and hierarchical clustering analysis

Cross validation was used to quantitatively finalize the pivotal diagnostic gene panel and assess its predictive power in breast cancer subtyping according to the status of ER, PR and HER2. Leave-1-out and 10-fold cross validations were used, where support vector machine (SVM) and *k*-nearest neighbor (KNN) were employed as the kernels. Both SVM and KNN are supervised machine learning methods widely applied in classification. SVM constructs a set of hyperplanes in a high-dimensional space, and the classification is achieved by the hyperplane that has the largest distance to the nearest training data point of any class. KNN classifies an object by taking a vote of its ‘*k*’ nearest neighbors, and the object is assigned to the class voted by the majority of the ‘*k*’ neighbors (*k* = 10 to be consistent with^34^). The statistics computed from 1000 simulations were reported.

The hierarchical clustering was used to draw heatmaps for the finalized diagnostic gene panel using R (https://www.r-project.org), where the distance matrix and agglomeration method were optimized to produce the optimal results.

We benchmark the predictive power of the pivotal gene panel against that in ref. 34 where GSE24450, TCGA and GSE22220 were used. As *FAM134B* is missing from GSE22220, we included GSE24450 and TCGA in this study. In addition, we added GSE25055 to generalize the subtyping functionality of the pivotal diagnostic gene panel. GSE24450 and GSE25055 were retrieved from the Gene Expression Omnibus (GEO) database. GSE24450 contains 183 primary breast tumors that were processed and hybridized to Illumina HumanHT-12_V3 Expression BeadChips. GSE25055 data was obtained using Affymetrix Human Genome U133A Array (HG-U133A) and encompasses 300 samples where 10 samples without consensus subtyping between immunohistochemistry marker-based and PAM50 classification were removed (original sample size is 310). TCGA data (level 3) was retrieved from the TCGA portal at http://tcga.cancer.gov/dataportal, which contains 451 samples profiled using Agilent 244 K Custom Gene Expression G4502A-07-3.

#### Patient tumor sample stratification

We performed tumor sample stratification based on the expression of each of the four pivotal diagnostic genes using GSE24450, TCGA and GSE25055 datasets. Student t test was used to assess the significance of each gene in distinguishing breast cancer subtypes stratified by ER, PR and HER2.

### Evaluation of diagnostic genes

#### Pathway and Gene Oncology enrichment analysis

Enrichment analyses on the pathways and Gene Oncology (GO) of the identified diagnostic genes were performed using Enrichr (http://amp.pharm.mssm.edu/Enrichr/). The performance of the enrichment analysis was evaluated by p-value, adjusted p-value, Z-score and C-score. The p-value is computed from the Fisher exact test which assumes a binominal distribution and independence of genes under test. The adjusted p-value is the p-value corrected from multiple hypotheses testing using the Benjamini-Hochberg method. The Z-score is computed as the deviation from the expected rank, which has been precomputed using Fisher’s exact test for many random input gene lists for each term in the gene set library. Combined score (denoted as ‘C-score’) was computed to assess the enrichment of each pathway or GO term using Equation 5

5 $${\rm{C}}={\mathrm{log}}_{10}({\rm{p}})\times {\rm{Z}},$$

where C is the C-score, p and Z each refers to the p-value and Z-score, respectively.

A gene set is a group of genes sharing a common biological function and used as the prior biological knowledge to be compared against for the enrichment analysis. Enrichr contains 103 gene sets, with genes covered in each set ranging from 280 to 49238.

In the pathway enrichment analysis, ‘BioCarta_2016’ was chosen as the gene set, where BioCarta is an interactive on-line resource designed for life science research with pathway information retrieval as a featured functionality^41^. In GO analysis the latest gene ontology annotations (‘GO_2015’) were used as the background.

#### Cancer proliferation marker enrichment analysis

Enrichment analysis of genes present in the integrated diagnostic network among cancer cell proliferation markers was conducted using Enrichr, where ‘Achilles_fitness_decrease’ was selected as the gene set. The Achilles project performed a genome-scale screen across 216 cancer cell lines for genes required for cancer cell proliferation and/or viability^42^.

### Data availability

The datasets analysed during the current study include 7 gene expression datasets, GSE70947, GSE15852, GSE20711, GSE65212, GSE18229-GPL887, GSE65194, GSE24450, retrieved from GEO (http://www.ncbi.nlm.nih.gov/geo), the level 3 breast cancer patient data downloaded from the TCGA repository (https://cancergenome.nih.gov), protein interaction data obtained from BioGRID (https://thebiogrid.org/), patient gene expression and clinical survival information stored in Kaplan Meier Plotter (http://kmplot.com/analysis/index.php?p=service&cancer=breast), pathways from BioCarta (https://cgap.nci.nih.gov/Pathways/BioCarta_Pathways), gene ontologies from GO database (http://www.geneontology.org/page/download-annotations), and proliferation markers identified from the Achilles project (https://portals.broadinstitute.org/achilles).

## Results

**Figure 1:**
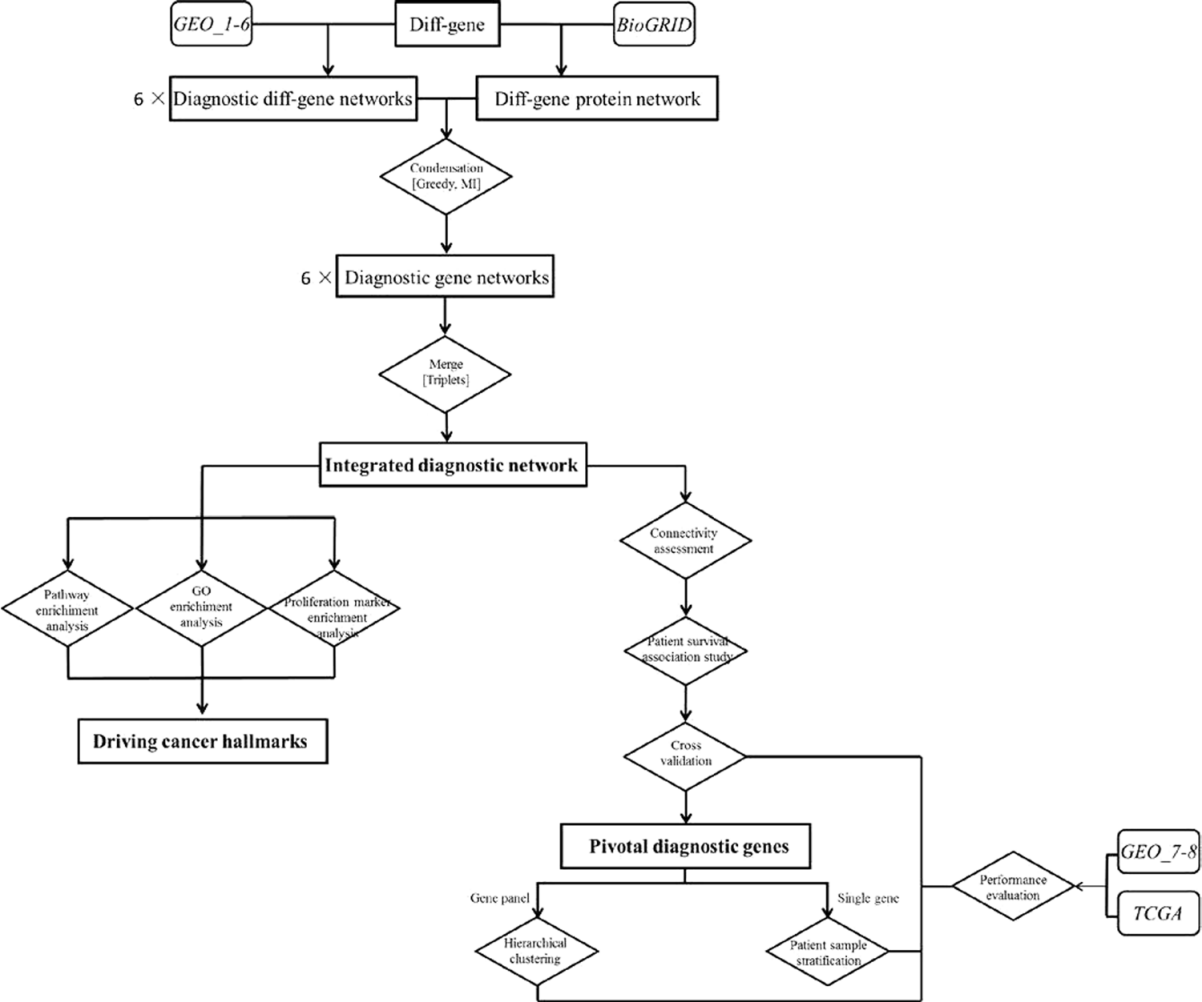
Workflow of this project. Each rounded square box represents one dataset, each square box shows one set of results, and each diamond box illustrates one operation together with associated algorithms. Datasets are shown in italic, where ‘*Data_1-6*’ represents GSE70947, GSE15852, GSE20711, GSE65212, GSE18229-GPL887, GSE65194, GSE24450, ‘*Data_7-9*’ represents GSE24450, GSE25055, TCGA, and ‘BioGRID’ means the BioGRID database. The primary outputs are highlighted in bold face, ‘6×’ means that 6 sets of networks were generated. Square brackets in each diamond box represent the algorithm or approach used in the operation.

The workflow of this study is summarized in Fig. 1.

### Diff-gene protein network

The diff-gene protein network, constructed by retrieving protein interactions from BioGRID using diff-genes identified in ref. 34, is comprised of 317 edges and 318 nodes and densely connected around two hubs, i.e., APP and ER (Supplementary Figure S1). The number of edges (degree) connected to APP and ER are 100 and 21, respectively. Those of APP and ER from the whole network stored in BioGRID are 2346 and 571, respectively.

### Diagnostic diff-gene networks

Six diagnostic diff-gene networks, each formed by mapping clinical gene expression data to the diff-gene protein network, were obtained (Supplementary Figure S2). These networks were named by concatenating the gene expression dataset with ‘PPI’, which represents protein interactions retrieved from BioGRID, by ‘&’. Each network contains, on average, 48 nodes and 53 edges, with detailed information available in Supplementary Table S1.

### Integrated diagnostic network

**Figure 2:**
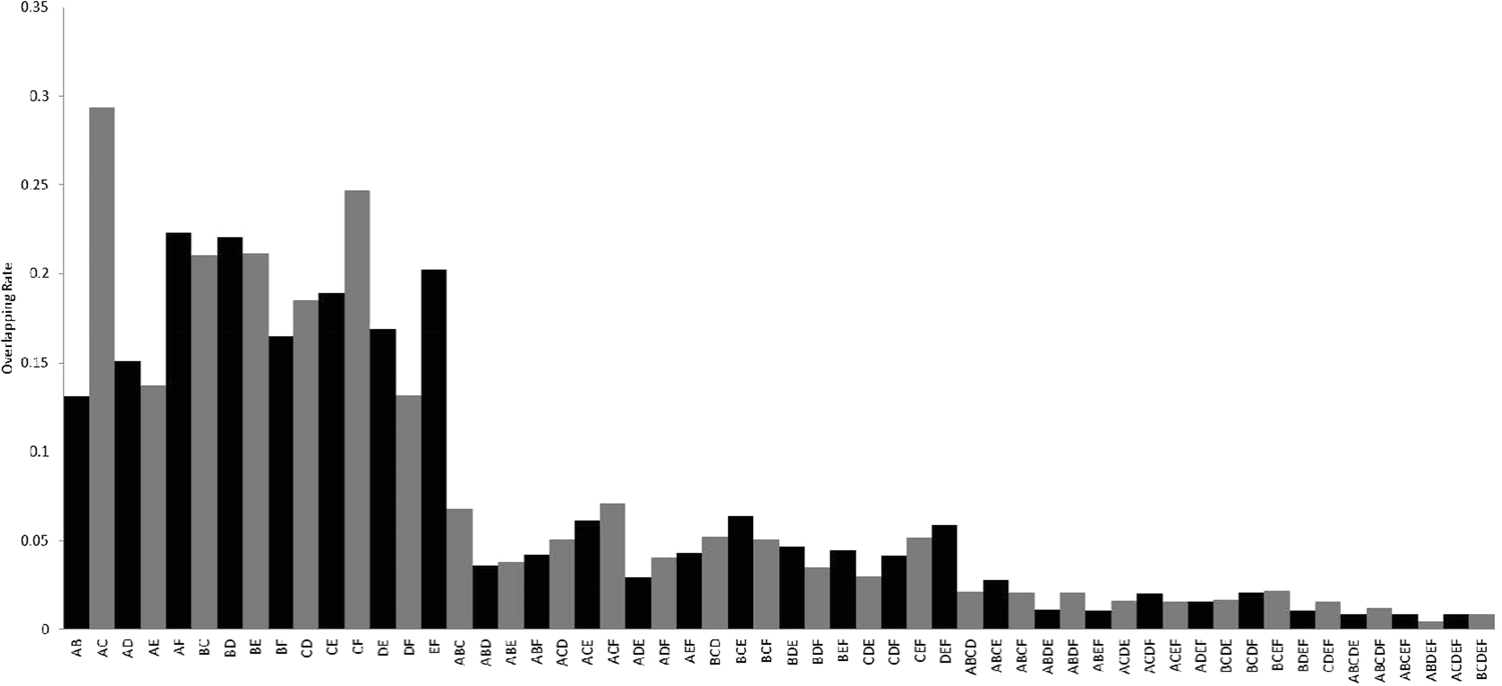
Overlapping rates for different combinations of diagnostic networks. (**A**,**B**,**C**,**D**,**E** and **F**) each denotes the diagnostic network GSE70947&PPI, GSE18229&PPI, GSE15852&PPI, GSE20711&PPI, GSE65194&PPI and GSE65212&PPI, respectively, where the network names are defined as the gene expression dataset concatenated with ‘PPI’ (representing BioGRID) by ‘&’.

The overlaping rates among diagnostic networks enter the plateau when we start merging them in triplets (Fig. 2), i.e., the double, triple, quadruple, quintuple, and sextuple integated networks contain, on average, 43, 42, 12, 8 and 1 genes, respectively.

**Figure 3:**
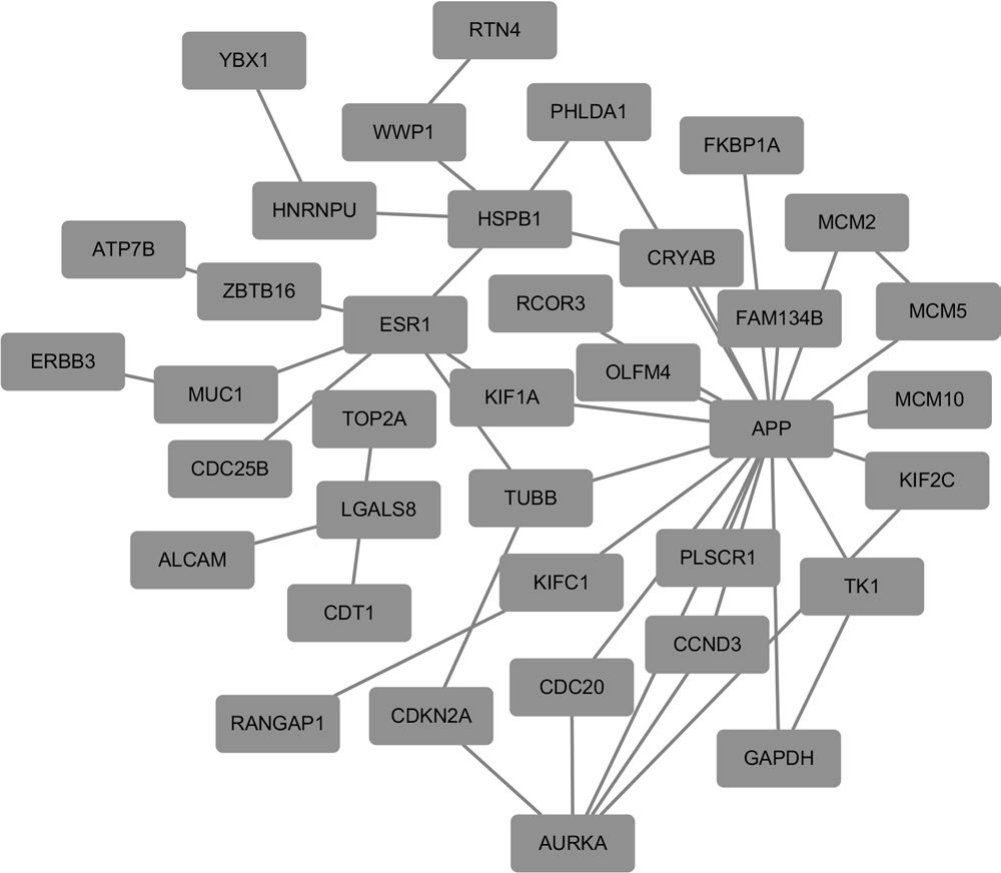
Integrated diagnostic network. This network was obtained by merging nodes and edges present in at least three diagnostic networks.

We, thus, selected nodes and edges at least present in three diagnostic networks and merged them as the integrated diagonosis network (Fig. 3), which includes 37 genes and 43 interactions. The condensed network preserves the two hubs (*APP* and *ER*) of the diff-gene protein network, with the degree being 19 and 6, respectively, for each gene.

Genes fell into <25, 25–50, 50–75 and >75 percentile of total degree represent 0.07%, 0.95%, 1.55% and 3.64% of total genes stored in BioGRID. The gene with the highest degree enrichement is *FAM134B* (33.33%) which together with *KIF2C* (28.57%), *ALCAM* (25%) and *KIF1A* (25%) represent the top 10 percentile degree enrichment among the 37 genes in the integrated diagnostic network.

### Pivotal diagnostic gene

#### Connectivity assessment

**Figure 4:**
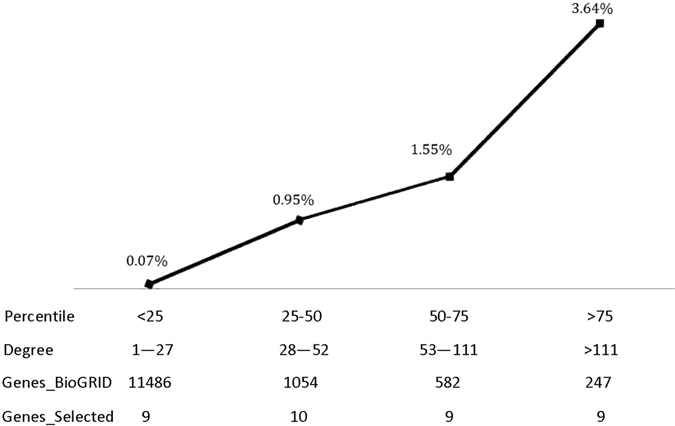
Enrichment of nodes degree in each percentile level for genes in the integrated diagnostic network. The percentile levels were defined as <25, 25–50, 50–75, and >75 percentile of the degrees of each gene in the integrated diagnostic network, which correspond to 1–27, 28–52, 53–111, and >111 number of degrees, respectively. ‘Genes_BioGRID’ represents the number of genes from BioGRID felt into a given percentile level of node degree, and ‘Genes_Selected’ shows that from the integrated diagnostic network.

The BioGRID database contains 13369 nodes and 109670 edges after removing singletons, with the degree of a single gene ranging from 1 to 3576. In accordance with the <25%, 25–50%, 50–75% and >75% percentile of degrees in the integrated diagnostic network, the number of degrees are classified into four groups, i.e., 1–27, 28–52, 53–111 and >111 degrees, respectively. Genes in the integrated diagnostic network are condensed in the group representing the top 25 percentile degrees, i.e., 3.64% of the total genes from BioGRID in this group as compared with the 1.55%, 0.95%, 0.07% statistics in the lower 25 percentile, 25 to 50 percentile and 50 to 75 percentile groups (Fig. 4). Permutation test with 1000 runs show that the high enrichment (3.64%) of the highly connected group (>75% percentile degree) in the integrated diagnostic network is not obtained by chance (p = 0.005).

The connectivity enrichment of each gene in the integrated diagnostic network as compared with the whole protein interaction network from BioGRID ranges from 33.3% (*FAM134B*) to 0.81% (*APP*), as listed in Supplementary Table S2. There are two break points, i.e., the 4^th^ and 6^th^ genes, where the connectivity enrichment of the diagnostic genes significantly drops (Supplementary Figure S3). The 3^rd^ and 4^th^ genes share the same connectivity enrichment. We, thereby, consider the top 6, top 5, top 4 and top 3 as candidates in the pivotal gene panel.

#### Patient survival association study

**Figure 5:**
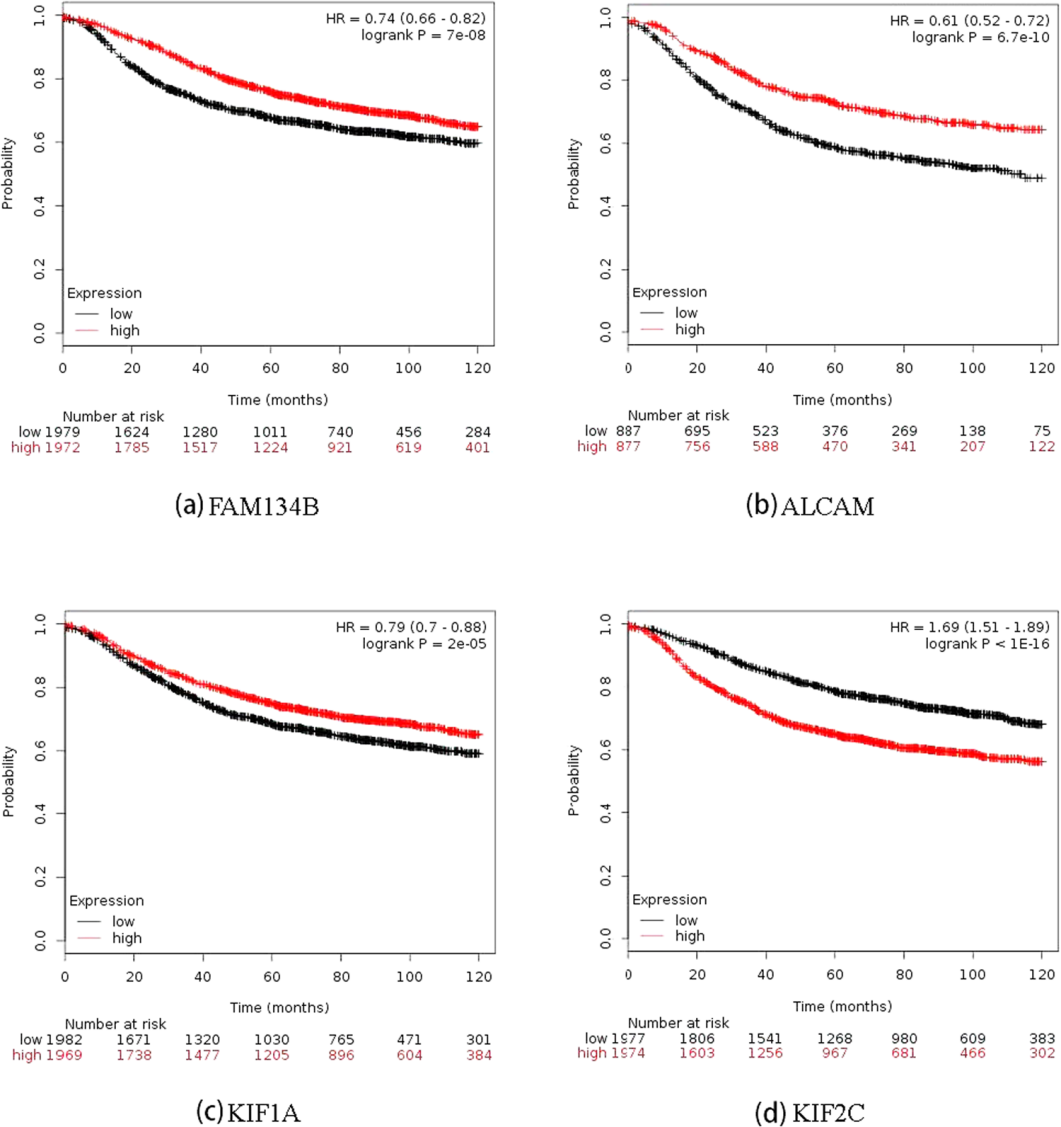
Breast cancer patient 10-year relapse free survival associated with each of the four pivotal diagnostic genes computed from Kaplan Meier Plotter.

The top five diagnostic genes are significantly associated with breast cancer 10-year relapse free survival (Fig. 5). *FAM134B* (p = 7E-08, HR = 0.79), *ALCAM* (p = 6.7E-10, HR = 0.61), *KIF1A* (p = 2E-05, HR = 0.79) confer protective effect, and *KIF2C* (p < 1E-16, HR = 1.69) and *KIFC1* (p < 1E-16, HR = 1.69) are risky on patient clinical outcome. No statistical significance was observed for *PHLDA1*. Thus, we exclude the 6^th^ gene from the candidate gene panel.

#### Cross validation

Leave-1-out cross-validation results show the maximum prediction power of 74.8% and 76.5% accuracies from 1000 runs as assessed by SVM and KNN (k = 10), respectively, when applied to the GSE24450 data; exhibit 68.1% (SVM) and 67.2% (KNN) accuracies when the TCGA dataset was used; and obtain 89.6% (SVM) and 88.7% (KNN) scores when GSE25055 was used (Table 1). The average behaviors are 67.8% (SVM) and 66.7% (KNN) using GSE24450; 58.6% (SVM) and 56.4% (KNN) using TCGA; and 77.6% (SVM) and 74.6% (KNN) using GSE25055 (Table 1).

**Table 1:** Cross-validations of the four pivotal diagnostic genes in differentiating breast cancer subtypes.

Gene panel	Statistics	GSE24450				TCGA				GSE25055			
		Leave-1-out		10-fold		Leave-1-out		10-fold		Leave-1-out		10-fold	
		SVM	KNN	SVM	KNN	SVM	KNN	SVM	KNN	SVM	KNN	SVM	KNN
3-gene panel	Median	0.677	0.670	0.677	0.670	0.565	0.539	0.579	0.544	0.739	0.713	0.731	0.716
	Mean	0.676	0.666	0.676	0.665	0.572	0.543	0.585	0.550	0.732	0.712	0.731	0.712
	Max	0.739	0.757	0.730	0.739	0.672	0.661	0.682	0.665	0.887	0.878	0.878	0.875
	Min	0.617	0.574	0.626	0.583	0.523	0.470	0.505	0.456	0.557	0.478	0.550	0.491
4-gene panel	Median	0.678	0.670	0.678	0.670	0.582	0.561	0.591	0.562	0.783	0.748	0.775	0.760
	Mean	0.678	0.667	0.678	0.667	0.586	0.564	0.596	0.561	0.776	0.746	0.776	0.757
	Max	0.748	0.765	0.748	0.765	0.681	0.672	0.690	0.682	0.896	0.887	0.889	0.878
	Min	0.626	0.591	0.635	0.591	0.525	0.479	0.522	0.483	0.565	0.478	0.587	0.528
5-gene panel	Median	0.678	0.670	0.678	0.670	0.573	0.548	0.586	0.557	0.765	0.739	0.749	0.745
	Mean	0.677	0.668	0.677	0.667	0.579	0.553	0.590	0.559	0.760	0.735	0.751	0.743
	Max	0.748	0.757	0.739	0.739	0.672	0.667	0.682	0.665	0.878	0.870	0.871	0.871
	Min	0.617	0.591	0.635	0.591	0.528	0.490	0.517	0.475	0.548	0.513	0.554	0.524

Leave-1-out and 10-fold represent two types of cross-validations used for performance assessment. ‘SVM’ and ‘KNN’ are used as the kernels for cross-validation, which represents support vector machine and *k*-nearest neighbor classifiers (*k* = 10), respectively. Statistics of 1000 rounds of iterations are shown.

Using 10-fold cross-validation and as compared with the leave-1-out approach, the same maximum and average prediction power were obtained using GSE24450; similar maximum and average scores were obtained using GSE25055, i.e., 88.9% (SVM) and 87.8% (KNN) for the maximum prediction power and 77.6% (SVM) and 75.7% (KNN) for the average performance; slightly higher performance was observed using TCGA data, i.e., 59.6% (SVM) and 56.1% (KNN) for the average performance, and 69% (SVM) and 68.2% (KNN) for the maximum behavior.

Results using SVM as the kernel are more stable than those using KNN as, in most cases, higher average performance, lower maximum and higher minimum values were obtained using SVM than KNN. 10-fold cross validation behaves better than the leave-1-out approach when data of relatively larger sample size was used. That is, the advantage of SVM over KNN becomes evident when TCGA data was used which encompasses 451 samples whereas GSE24450 and GSE25055 have 183 and 300 samples, respectively.

Most statistics measured for the 4-gene panel outweigh those in the 3-gene and 5-gene panels, though the difference is nuance (Table 1). Using GSE24450 as the discovery set for finalizing the pivotal gene panel, we selected the 10-fold cross validation approach (with SVM being the kernel) to assess the trajectory of the prediction power of the gene panels where one gene from the integrated diagnostic network was added at one time. The results show that 1) having more genes added in the panel, overall, improves the prediction power, and 2) the trajectory undergoes a sharp increase during the first 4 genes followed by a mild recession and relativley long plateau (Supplementary Figure S4). We thus consider the 4-gene panel as pivotal genes for the subsequent analyses and discussions.

#### Hierarchical clustering analysis

**Figure 6:**
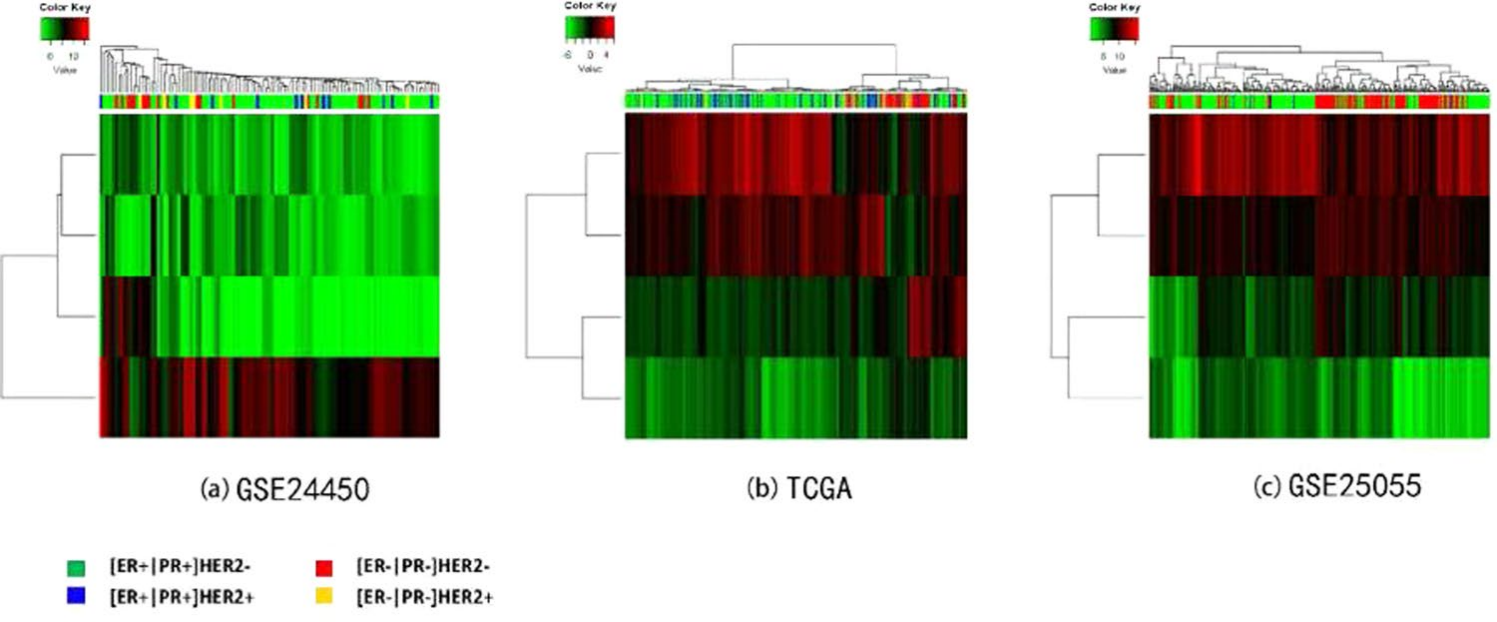
Breast cancers from GSE24450, TCGA and GSE25055 clustered by the four pivotal diagnostic genes.

Four subtypes, [ER+|PR+]HER2−, [ER+|PR+]HER2+, [ER−|PR−]HER2+, [ER−|PR−]HER2- (also named TNG), were defined based on the status of ER, PR and HER2, conventionally used in clinic. Using only the four pivotal diagnostic markers, ER- tumors (red and yellow), especially the [ER−|PR−]HER2− cohort (red), could be clearly distinguished from ER+ samples (green and blue) (Fig. 6) using GSE24450, TCGA and GSE25055 datasets.

#### Patient sample stratification

**Figure 7:**
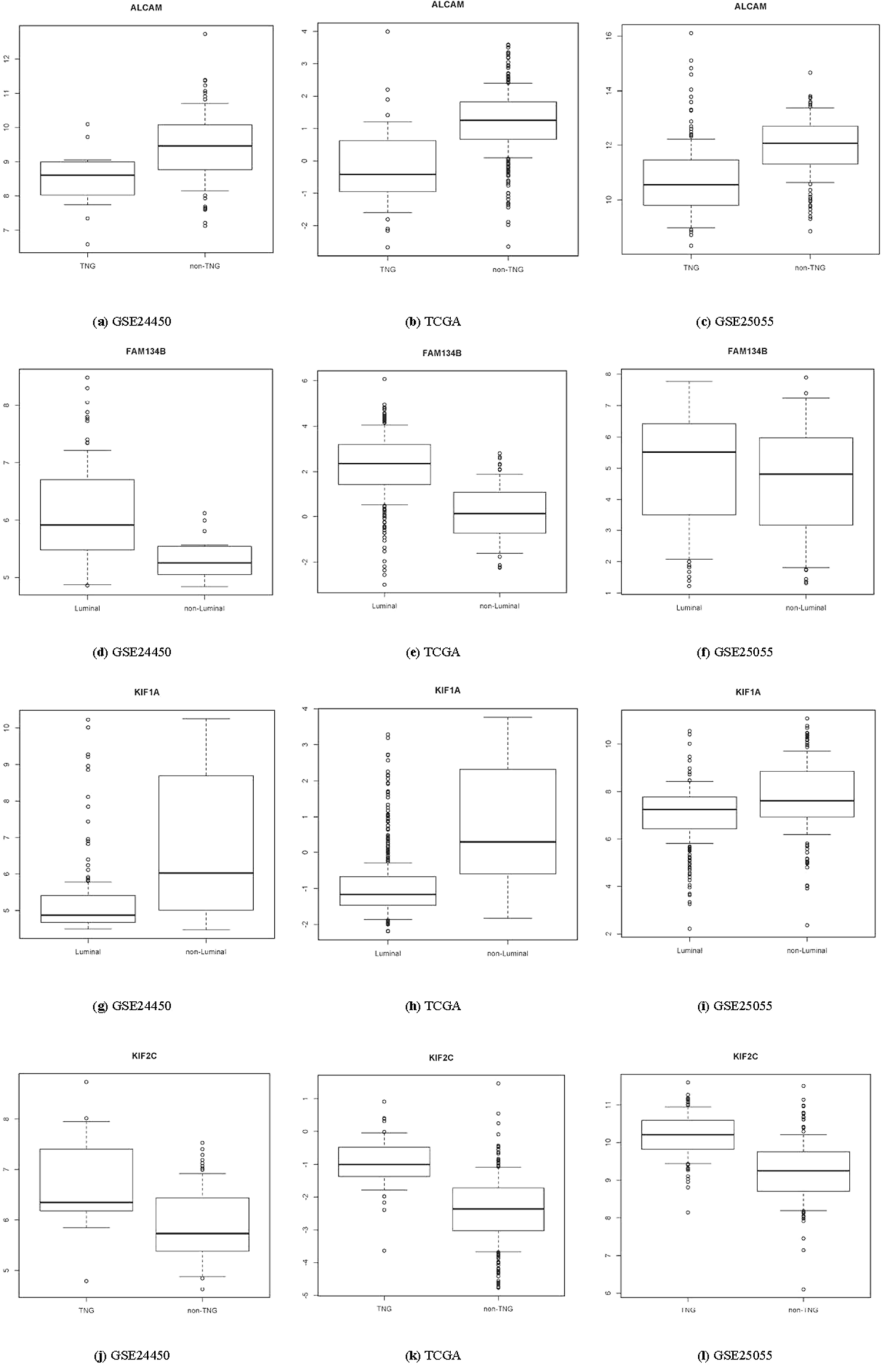
Breast cancers from TCGA categorized into ‘luminal vs. non-luminal’ or ‘TNG vs. non-TNG’ for each of the four pivotal diagnostic genes.

Among the four genes, *FAM134B* and *KIF1A* function in differentiating ER positive and ER negative subtypes. The p values are 3.10E-25 (*FAM134B*) and 3.66E-13 (*KIF1A*) using TCGA data; 2.71E-10 (*FAM134B*) and 4.83E-03 (*KIF1A*) using GSE24450; and 6.33E-03 (*FAM134B*) and 1.43E-05 (*KIF1A*) using GSE25055 (Fig. 7). *ALCAM* and *KIF2C* could nicely distinguish TNG from the rest. That is, the p values are 1.01E-12 (*ALCAM*) and 2.24E-21 (*KIF2C*) using TCGA data; 1.82E-04 (*ALCAM*) and 1.97E-03 (*KIF2C*) using GSE24450; and 4.11E-14 (*ALCAM*) and 2.24E-27 (Fig. 7).

### Diagnostic genes

#### Pathway enrichment analysis

Genes from the integrated diagnostic network are enriched in 22 pathways obtained from BioCarta^41^ (Supplementary Figure S5, Supplementary Table S3). The C-score and the p-value decreases and increases dramatically from the 6^th^ enriched pathway (Supplementary Figure S5). The top five pathways are ‘CDK regulation of DNA replication’ (p = 7.15E-05, C-score = 14.54), ‘downregulation of MTA3 in ER-negative breast tumors’ (p = 7.15E-05, C-score = 11.11), ‘role of HER2 in signal transduction and oncology’ (p = 5.37E-03, C-score = 8.72), ‘cyclines and cell cycle regulation’ (p = 4.52E-03, C-score = 6.07) and ‘role of Ran in mitotic spindle regulation’ (p = 1.33E-03, C-score = 5.85) (Table 2). Three out of the 5 pathways are associated with cell cycle, one represents the metastatic feature of ER negative subtype, and one shows the importance of HER2 mediated signaling in differentiating breast cancer subtypes.

**Table 2:** Statistics of the top 5 pathways enriched by genes present in the integrated diagnostic network.

Pathways	Adjusted p-value	Z-score	C-score	Genes
CDK Regulation of DNA Replication	7.15E-05	−1.5235	14.54	*CDT1;MCM5;MCM2*
Downregulation of MTA3 in ER-negative Breast Tumors	7.15E-05	−1.1643	11.11	*HSPB1;ESR1;GAPDH*
Role of HER2 in Signal Transduction and Oncology	5.37E-03	−1.6685	8.72	*ERBB3;ESR1*
Cyclins and Cell Cycle Regulation	4.52E-03	−1.1252	6.07	*CCND3;CDKN2A*
Role of Ran in mitotic spindle regulation	1.33E-03	−0.8836	5.85	*RANGAP1;AURKA*

Genes from the integrated diagnostic network and enriched in a given pathway are listed accordingly as ‘Genes’.

#### Gene Ontology enrichment analysis

**Figure 8:**
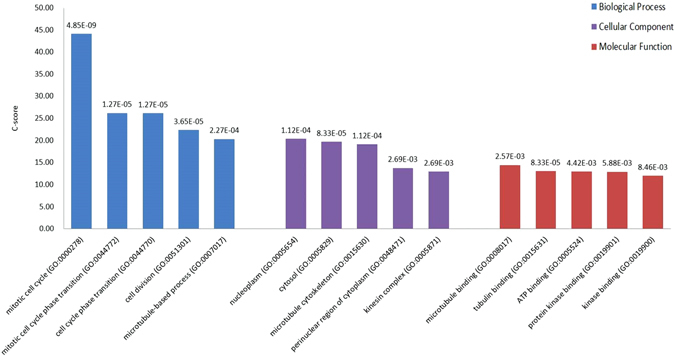
Top 5 enriched GO terms for genes in the integrated diagnostic network.

72 biological processes, 17 cellular components and 14 molecular functions, collectively called GO terms, are enriched by genes from the integrated diagnostic network with adjusted p values below 0.05 (Supplementary Table S4). The top 5 enriched biological processes are ‘mitotic cell cycle’ (adjusted p = 4.85E-09, C-score = 44.12), ‘mitotic cell cycle phase transition’ (adjusted p = 1.27E-05, C-score = 26.19), ‘cell cycle phase transition’ (adjusted p = 1.27E-05, C-score = 26.14), ‘cell division’ (adjusted p = 3.65E-05, C-score = 22.33), and ‘microtubule-based process’ (adjusted p = 2.27E-04, C-score = 20.32), which are all associated with cell cycle and division (Fig. 8). The top 5 enriched cellular components are ‘nucleoplasm’ (adjusted p = 1.12E-04, C-score = 20.37), ‘cytosol’ (adjusted p = 8.33E-05, C-score = 19.70), ‘microtubule cytoskeleton’ (adjusted p = 1.12E-04, C-score = 19.12), ‘perinuclear region of cytoplasm’ (adjusted p = 2.69E-03, C-score = 13.74) and ‘kinesin complex’ (adjusted p = 2.69E-03, C-score = 12.91), which are locations and components involved during mitotic cell division (Fig. 8). Accordingly, the top 5 enriched molecular functions, ‘microtubule binding’ (adjusted p = 2.57E-03, C-score = 14.41), ‘tubulin binding’ (adjusted p = 4.42E-03, C-score = 13.04), ‘ATP binding’ (adjusted p = 4.42E-03, C-score = 12.94), ‘protein kinase binding’ (adjusted p = 5.88E-03, C-score = 12.91) and ‘kinase binding’ (adjusted p = 8.46E-03, C-score = 11.99), convolve the proteins, ATP and kinases required for cell division (Fig. 8).

#### Cancer proliferation marker enrichment analysis

Out of the 216 cell lines used to screen genes having a context-specific effect on cell proliferation and/or viability in the Achilles project^42^, 64 are enriched with genes in the integrated diagnostic network (Supplementary Figure S6. The C-score drops considerably and the p value undergoes a sharp increase from the 6^th^ cell line (cell lines are ranked with the decrease of the C-score and increase of the p-value). The top five cell lines are ZR7530, SNU840, HCC2218, NCIH23 and BT474, among which 4 out of 5 are breast or ovary cancers. Genes enriched in these 5 cell lines are *TOP2A*, *HER3*, *CDC25B*, *MCM2*, *TUBB*, *HNRNPU*, and *CCND3*, where *TOP2A*, *HER3*, *CC25B* and *MCM2* appear three times, *TUBB* and *HNRNPU* pop up twice and *CCND3* is only present in the ovary cell line SNU840 (Table 2). Genes enriched in the top breast cancer cell lines are *TOP2A*, *HER3*, *CDC25B*, *MCM2* and *TUBB* (Table 2).

## Discussion

### Integrated diagnostic network reveals 4 pivotal genes with diagnostic potential

The integrated diagnostic network preserves the top two hubs of the diff-gene protein network retrieved from BioGRID, i.e., *APP* and *ER* (Supplementary Figure S7). It is intuitive that ER dominates the diagnostic network given its prominent roles and canonical use in breast cancer subtyping^7^. APP, however, is even more promiscuous, which has 4 to 5 times number of edges of ER in the whole protein interaction network of BioGRID or the diff-gene protein network, and the fold drops to 3 when the network is condensed to the integrated diagnostic network. This, on one hand, implicates that the network, once trimed to capture breast cancer heterogeneity, is shifted towards ER-driven and, on the other hand, suggests the critical roles played by APP in mediating carcinogenesis and subtype differentiation. *APP* has multiple human isoforms due to alternative splicing and encodes a type I transmembrane protien (amyloid precursor protein) expressed in many tisues. APP has been implicated in many cellular processes including hormonal regulation^43^. In particular, *APP* has been reported as a primary androgen target gene promoting prostate cancer growth^43^, and suggested to promote breast cancer proliferation with its immunohistochemical status proposed as a prognostic factor in ER positive breast cancers^44^; a recent study further unveiled its role in accerlerating the motility of advanced breast tumors, implicating its therapeutic targeting opportunity^45^.

Genes with degrees over-represented in the integrated diagnostic network are *FAM134B*, *KIF2C*, *ALCAM* and *KIF1A*, the combined effort of which has shown a comparable subtyping accuracy with the 1015 diff-genes reported in ref. 34 (Fig. 6, Table 1). The leave-one-out cross validations using GSE24450 (namely HEBCS in ref. 34) were reported to be 0.757 and 0.748, respectively, from SVM and KNN in ref. 34, and were 0.75 and 0.77, respectively, in this study; similarly, 0.735 and 0.723 were obtained using TCGA data from SVM and KNN in ref. 34, and 0.67 was observed from both approaches here (Table 1). These results suggest that the four pivotal diagnostic genes capture, if not all, the majority of the subtyping information imbedded in the diff-genes. By varying subtype combinations, we found that *FAM134B* and *KIF1A* function best in stratefying cancers according to ER status, and *ALCAM* and *KIF2C* act as the identifiers of triple negative cancers; while *FAM134B* and *ALCAM* express relatively higher in ER+ or non-TNG subtypes, *KIF1A* and *KIF2C* have comparatively lower expression in tumors of these classes (Fig. 7). Expression of these four genes, thus, may offer a succint panel for breast cancer diagnosis in addition to ER, PR and HER2 status. Truly, in accordance with this, patient 10-year relapse free survival analysis of each gene from this panel reveals that over-expression of *FAM134B*, *ALCAM*, *KIF1A* and low-expression of *KIF2C* each conveys a favorable clinical outcome with statistical significance (Fig. 5). *FAM134B* encodes an endoplasmic reticulum-anchored autophagy receptor mediating the degration of endoplasmic reticulum^46^. Its genetic mutation, resulting in decreased *FAM134B* expression, is a frequent event in the progression of oesophageal squamous cells^47^ and colorectal cancers^48^, which is adversely associated with patient clinical and pathological parameters and congruent with the tumor suppressive properties of *FAM134B* as previously reported^48^ as well as demonstrated in this study (Figs 6 and 7). ALCAM, the activated leukocyte cell adhesion molecule, has been known involved in cell migration and adhesion^49, 50^, in accordance with its identified role here in distinguishing TNG breast cancers, featured by high invasiveness, from the rest (Fig. 7). Decreased *ALCAM* expression has been implicated in poor breast cancer prognosis and promoted metastasis ability^49–54^, confirming with its tumor suppressive roles observed in Fig. 5 as well as previously suggested^55^. Impaired *ALCAM* expression is associated with induced ER+ breast cancer cell apoptosis and autophagy^56^, and down-regulating *ALCAM* expression sensitizes ER+ breast cancers to Tamoxifen treatment^57^, suggesting the therapeutic potential of down-regulating *ALCAM* in ER+ cancers which is consistent with its relatively higher expression in such tumor subtypes (Fig. 7). Both *KIF1A* and *KIF2C* encode members of the kinesin family, whose active movement supports several cellular functions including mitosis^58^. *KIF1A* was reported over-expressed in ER- breast cancer cell lines MDA-MB-231 and MDA-MB-468, and contributes to their chemotherapeutic resistance^15^. Elevated level of *KIF2C* was found in non-small cell lung cancer cells, which promotes cancer cell migration and could be suppressed by targeting the RAS-RAF-MEK1 pathway^59^. These not only support our observations on their diagnostic potential (Figs 6 and 7) but also suggest their therapeutic opportunities in cancer control.

### Enrichment analysis reveals 5 cancer hallmarks driving breast cancer heterogeneity

The top 5 pathways enriched by genes in the integrated diagnostic network (adjusted p < 0.01) are ‘CDK regulation of DNA replication’, ‘down-regulation of MTA3 in ER negative breast cancers’, ‘role of HER2 in signal transduction’, ‘cyclins and cell cycle regulation’ and ‘role of Ran in mitotic spindle regulation’ (Table 3). These pathways show two prominent phenotypic features dominating breast cancer heterogeneities, i.e., proliferation and metastasis, and imply three cancer hallmarks. That is, three out of the five pathways reflect the ‘enabling replicative immortality’ (cell cycle) hallmark, one is associated with the ‘sustaining proliferative signaling’ (HER2 transduction), and one represents the ‘activating invasion & metastasis’ (MTA3 is metastasis associated 1 family member 3). As *MTA3* is an estrogen-regulated gene^60^ whose promoter region contains an ER binding site, these pathways also consolidate the roles of ER and HER2 in breast cancer subtyping.

**Table 3:** Top 5 enriched cell lines from cancer proliferation marker enrichment analysis.

Name	Type	p-value	Z-score	C-score	Genes
ZR7530	breast	0.023110909	−1.760811151	6.633768881	*TOP2A;HER3;CDC25B;MCM2*
SNU840	ovary	0.023110909	−1.713280168	6.454698254	*CCND3;TUBB;HNRNPU*
HCC2218	breast	0.023110909	−1.710427594	6.443951326	*TOP2A;HER3;CDC25B;MCM2*
NCIH23	lung	0.023110909	−1.67847526	6.323572486	*TOP2A;HNRNPU;MCM2*
BT474	breast	0.035418911	−1.619832446	5.411065504	*HER3;TUBB;CDC25B*

Cell line name, type, p-value, Z-score, C-score and genes enriched in each cell line are provided.

Genes enriched in these 5 pathways are *ER*, *HER3*, *MCM2*, *MCM5*, *CDT1*, *CCND3*, *CDKN2A*, *RANGAP1*, *AURKA*, *HSPB1*, *GAPDH*. Genes such as *ER* and *HER3* reflect the proliferative property of breast cancer cells. ER has long been recognized to mediate cell signaling in response to hormonal stimuli and known to drive the proliferative feature of breast cancer cells^61^. HER3 forms heterodimers with other members of this family, leading to the activation of pathways governing cell proliferation and differentiation. Seven of the 11 genes suggest the vital roles of the G1/S and G2/M check points for ‘enabling replicative immortality’. MCM2 and MCM5 are members of the MCM family of chromatin-binding proteins which, together with CDT1, are involved in DNA replication initiation and up-regulated during the G1/S transition. *CCND3* encodes cyclin D3 that forms a complex with CDK4/6, the activity of which is required for the G1/S transition in the cell cycle; and *CDKN2A* encodes an inhibitor of CDK4. *RANGAP1* encodes a protein interacting with Ras-related nuclear protein 1 (Ran), which is phosphorylated by the cyclin B/CDK1 complex (M phase kinase) and plays essential roles during cell mitosis^62^. AURKA is a cell cycle regulated kinase involved in microtubule formation and/or stabilization at the spindle pole during chromosome segregation and, thus, implicated with fundamental roles during mitosis and meiosis^63^. HSPB1, a member of the heat shock protein family, is reported to suppress *PTEN* level and, consequently, leads to reduced apoptosis in human breast cancer cells^64^, implicating the properties of cancer cells in ‘resisting cell death’. *GAPDH* encodes the glyceraldehyde-3-phosphate dehydrogenase whose up-regulation is correlated with aberrant gene profiling associated with both glycolysis and gluconeogenesis^65^. This suggests the Warburg effect, which represents the ‘deregulating cellular energetics’ hallmark.

Almost all genes enriched in the top 5 pathways have been implicated with cancer diagnostic potentials. ER has been canonically used as a clinical routine for breast cancer subtyping^7^. *HER3* overexpression has been observed in diverse human cancers and been reported diagnostic of poor outcome in, e.g., breast cancer^66^ and melanoma^67^. *MCM2* and *MCM5* have been used for the diagnosis of colon cancers^68^. The prognostic value of *CDT1* has been recently evaluated in breast cancer, whose over-expression was observed in tumor cells and significantly associated with poor patient survival^69^. *CCND3* amplification has been proposed as a marker predicting tumor progression in, e.g., breast cancer^70^ and bladder urothelial carcinoma^71^. *CDKN2A* hyper-methylation has been suggested as a predictive factor for unfavorable prognosis of, e.g., colorectal cancer^72, 73^, rectal cancer^74^, and adult acute lymphoblastic leukemia patients harboring *BCR-ABL1* fusions^75^. *AURKA* over-expression is reported strongly associated with tumor grade and proposed with prognostic value for disease progression^76^. *HSPB1* encodes the heat-shock protein 27 which plays crucial roles in tumorigenesis and is reported an independent prognosis marker for malignancies such as lung cancer^77^. Elevated level of *GAPDH* positively associated genes is proportional to the malignant stage of various tumors and unfavorable prognosis^65^.

Gene ontology analysis reveals cell division to be the most enriched cellular event differentiating breast cancer subtypes (Fig. 8). This, together with the 7 out of 11 genes identified from pathway analysis and participating directly in cell cycle, implicate that ‘enabling replicative immortality’ may be one of the driving hallmarks fostering the proliferative feature of breast cancer cells and their differentiation.

Cancer proliferation marker enrichment analysis reveals that 7 genes from the integrated diagnostic network are enriched in cancer cells. Among them, 5 (*TOP2A*, *HER3*, *CDC25B*, *MCM2* and *TUBB*) are from breast cancer cell lines (Table 3) and, in particular, HER2 positive cells (ZR7530 is [ER+PR−]HER2+, HCC2218 and BT474 are [ER−PR−]HER2+^78^). TOP2A, topoisomerase II alpha, functioning as an enzyme relaxing DNA supercoils, has long been used as a cancer proliferation marker and applied for breast tumor subtyping^7^. Importantly, abnormal *TOP2A* expression has been reported associated with increased cancer responsiveness to anthracycline-based chemotherapy^79^, suggesting its therapeutic implications besides confirmed diagnostic roles. *HER3* encodes an EGFR family protein that is used as a prognostic marker in hormone receptor-negative breast cancers including the TNG and the HER2 positive subtype^66, 67^, and is as critical as HER2 in cell proliferation maintenance^80^. CDC25B is a member of the CDC25 family of phosphatases that activates the cyclin dependent kinase CDC2 and required for the entry of cells into mitosis. The association between *CDC25B* expression and cell proliferation is multifaceted: on one hand, *CDC25B* is up-regulated in multiple tumor types with increased levels correlated with higher proliferation, and its elevated level in the mammary glands has led to accelerated mammary epithelial proliferation that ultimately leads to tumor formation when exposed to the carcinogen DMBA *in vivo* ^81^; on the other hand, its tumor suppressive roles and anti-proliferative effect have been reported by several studies^81, 82^. *MCM2* expression is correlated with that of *KI67*, a widely used proliferation marker in addition to ER, PR and HER2 for breast cancer subtyping in some studies^7^, and proposed as a sensitive maker of gastric cardiac cancer^83^. *TUBB* encodes the beta chain of tubulin, which polymerizes into microtubules that function in many essential cellular processes including mitosis, and thus indicative of cell proliferation. It is reported that targeting tubulin arrests mitosis and inhibits tumor cell proliferation, rendering microtubule-targeted drugs indispensable for the therapy of various cancers^84^. Some of these proliferation markers have intrinsic connections, so far reported, with *HER2* status or expression. For instance, *TOP2A* aberrations are frequently found in *HER2*-amplifed breast cancers, accounting for 30–90% of such tumors^7, 85^. HER3 forms heterodimers with HER2 in downstream signal transduction, and plays a central role in *HER2*-amplified breast cancers^80^. *CDC25B* expression could be induced through HER2 signal transduction in human lung cancer cells^86^. These, collectively, suggest the importance of ‘sustaining proliferative signaling’ and, in particular, HER2 transduction, in driving the complex morphological and pathological features of breast cancers.

## Conclusion

This study constructed an integrated diagnostic network composed of 37 nodes and 43 edges, by using information integrated from 6 publically available gene expression datasets and protein interactions retrieved from BioGRID to trim the 1015 diff-genes previously reported. We identified 4 pivotal diagnostic genes (*FAM134B*, *KIF2C*, *ALCAM*, *KIF1A*) from this network, which form a largely reduced gene panel preserving comparable subtyping efficacies with the initial 1015 diff-genes. Further pathway, GO, and proliferation marker enrichment analyses of the integrated diagnostic network collectively suggest two carcinogenic transitions governing breast cancer differentiation, i.e., proliferation and metastasis, and five out of 10 cancer hallmarks^87^, i.e., ‘enabling replicative immortality’ (i.e., cell cycle, especially G1/S and G2/M), ‘sustaining proliferative signaling’ (ER, HER2), ‘resisting cell death’, ‘deregulating cellular energetics’ (aerobic glycolysis), and ‘activating invasion & metastasis’ empowering such processes, with the first two being the most prominent. Our work provides a gene panel of reasonable size with clinical translation potential, and hallmarks driving breast cancer heterogeneities. The pivotal genes and primarily hallmarks (or implicated top pathways) identified may offer novel diagnostic markers or therapeutic targets, alone or in combination with current clinical modalities, for the benefit of breast cancer patients.

## Electronic supplementary material

supplementary information
