# Supplementary material for: Integrated diagnostic network construction reveals a 4-gene panel and 5 cancer hallmarks driving breast cancer heterogeneity
Source: Sci Rep. 2017 Jul 28;7:6827. doi: 10.1038/s41598-017-07189-6 (PMC5533795; doi:10.1038/s41598-017-07189-6)
Supplement: Supplementary file 1 — supplementary information [file 41598_2017_7189_MOESM1_ESM.pdf]

Integrated diagnostic network construction reveals a 4-gene panel and 5 cancer hallmarks driving breast cancer heterogeneity

Xiaofeng Dai[a,b*], Tongyan Hua[a,b], Tingting Hong[c]

[a]National Engineering Laboratory for Cereal Fermentation Technology, Jiangnan University, Wuxi, China

[b]The Key Laboratory of Industrial Biotechnology, Ministry of Education, School of Biotechnology, Jiangnan University, Wuxi, China

[c]Department of medical oncology, the affiliated hospital of Jiangnan University, the fourth people's hospital of Wuxi, Wuxi, China.

* Corresponding author:
Xiaofeng Dai: 1281423490@qq.com

Supplementary Figures

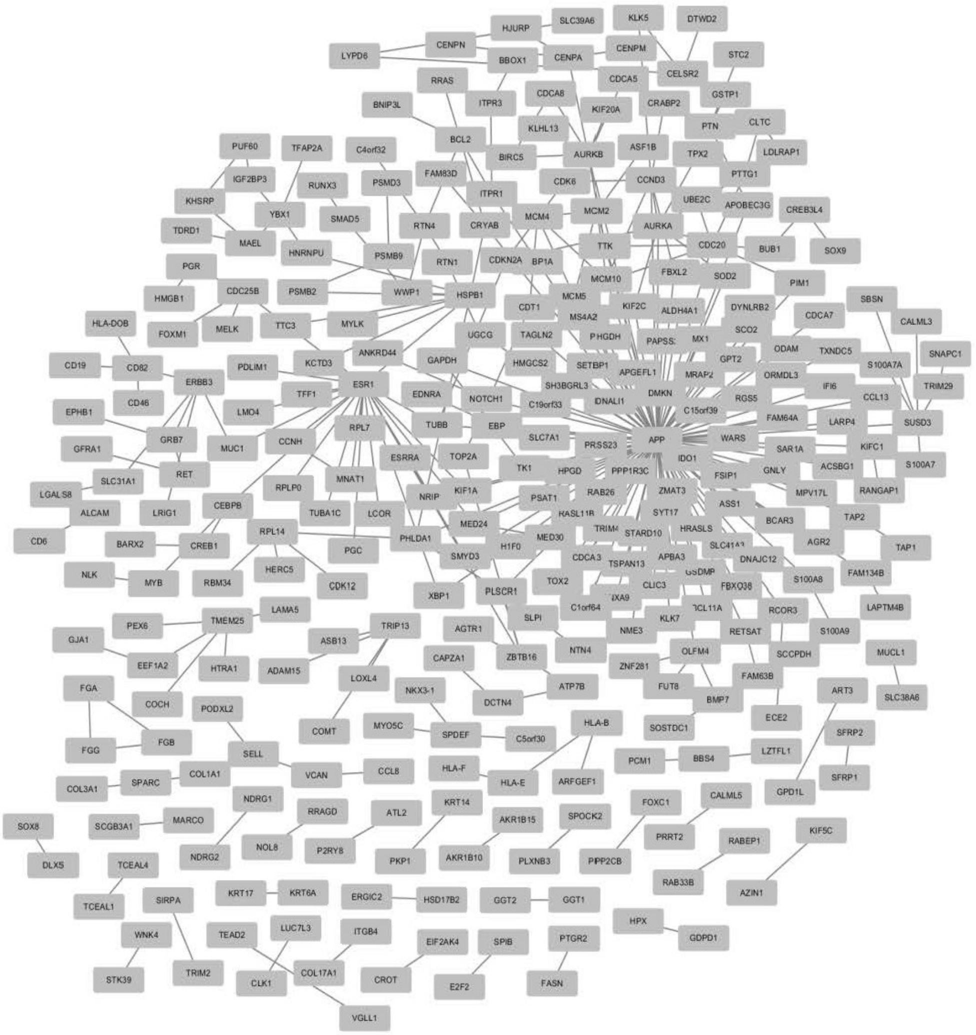

Supplementary Figure S1. Diff-gene protein network. This is constructed by retrieving protein interactions of the 1015 diff-genes from BioGRID.

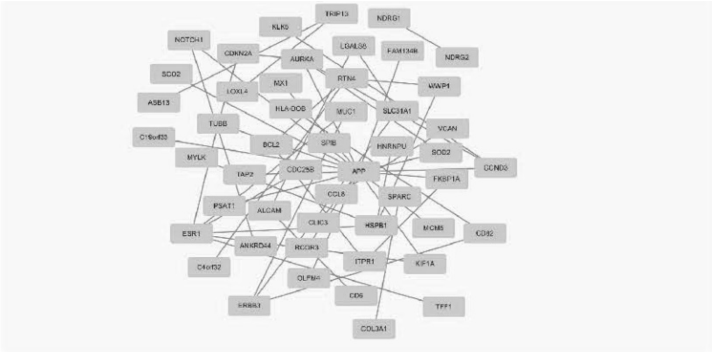

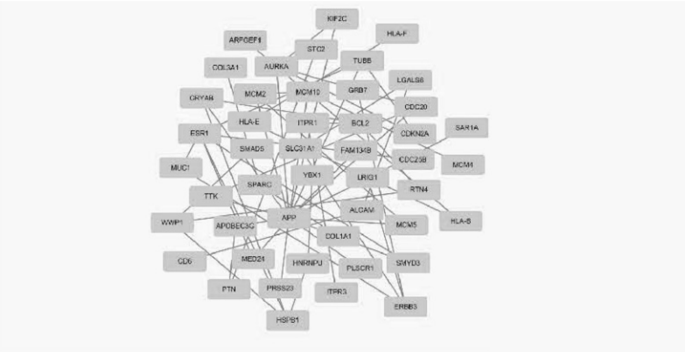

(a) GSE70947&PPI

(b) GSE15852&PPI

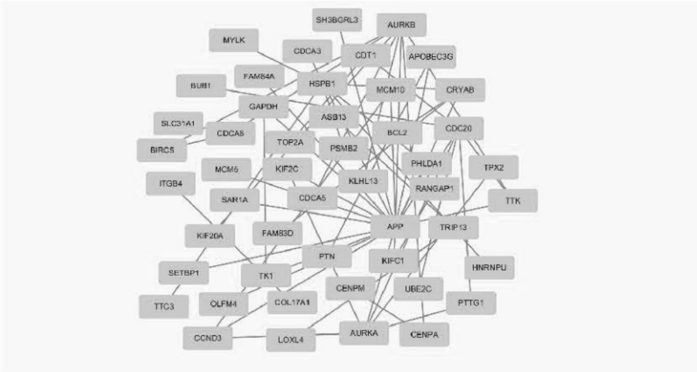

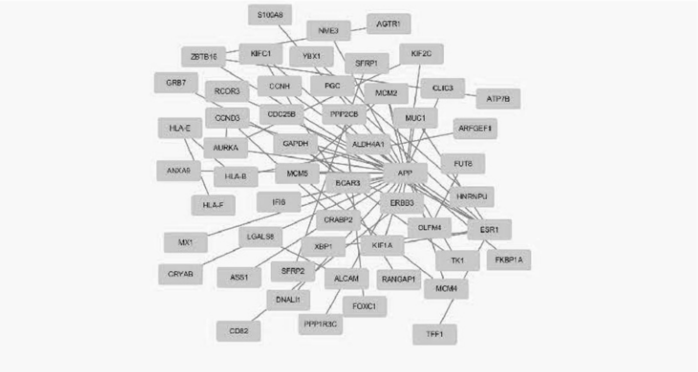

(c) GSE20711&PPI

(d) GSE65212&PPI

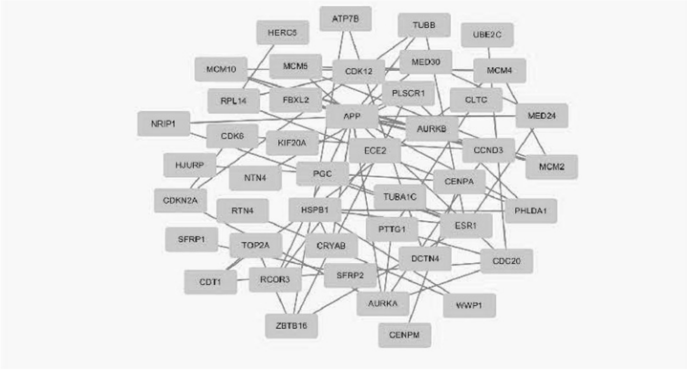

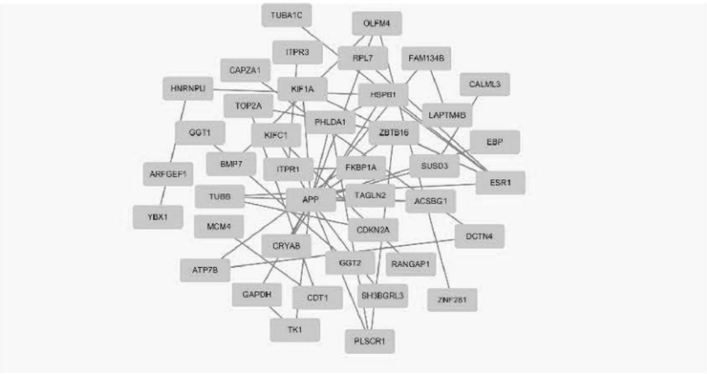

(e) GSE18229&PPI

(f) GSE65194&PPI

Supplementary Figure S2. Diagnostic networks. The network names are defined as the gene expression data set concatenated with ‘PPI’ (representing the protein interaction network retrieved from BioGRID) by ‘&’.

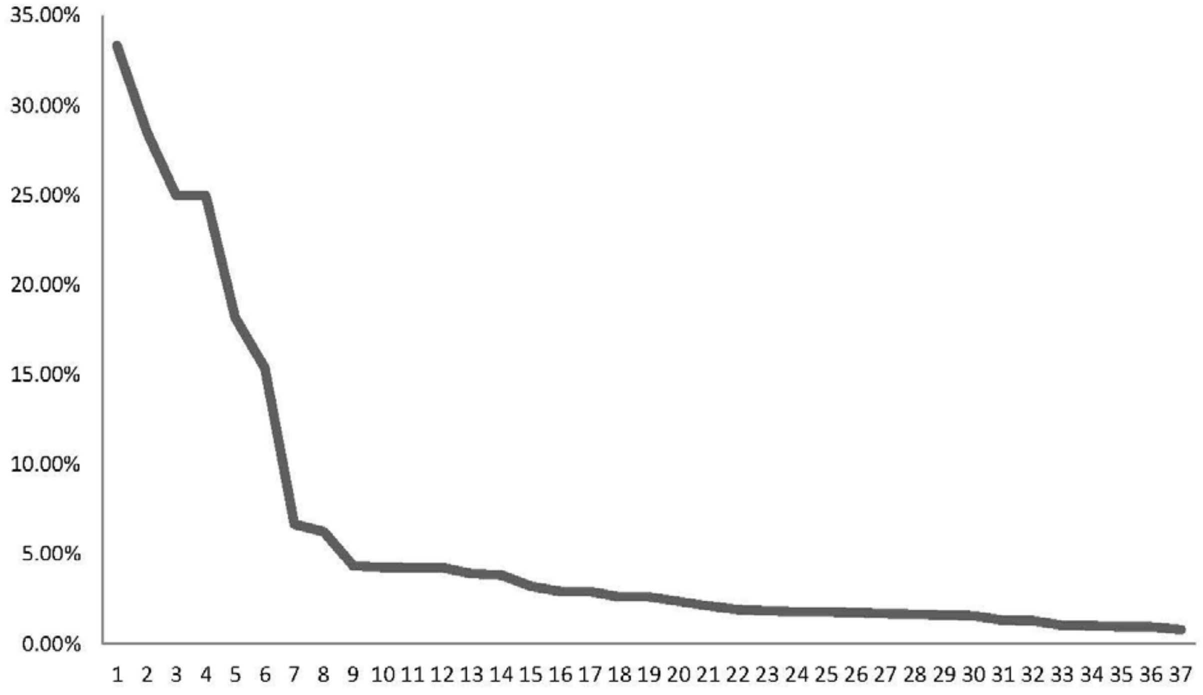

Supplementary Figure S3. Connectivity enrichment of genes in the integrated diagnostic network

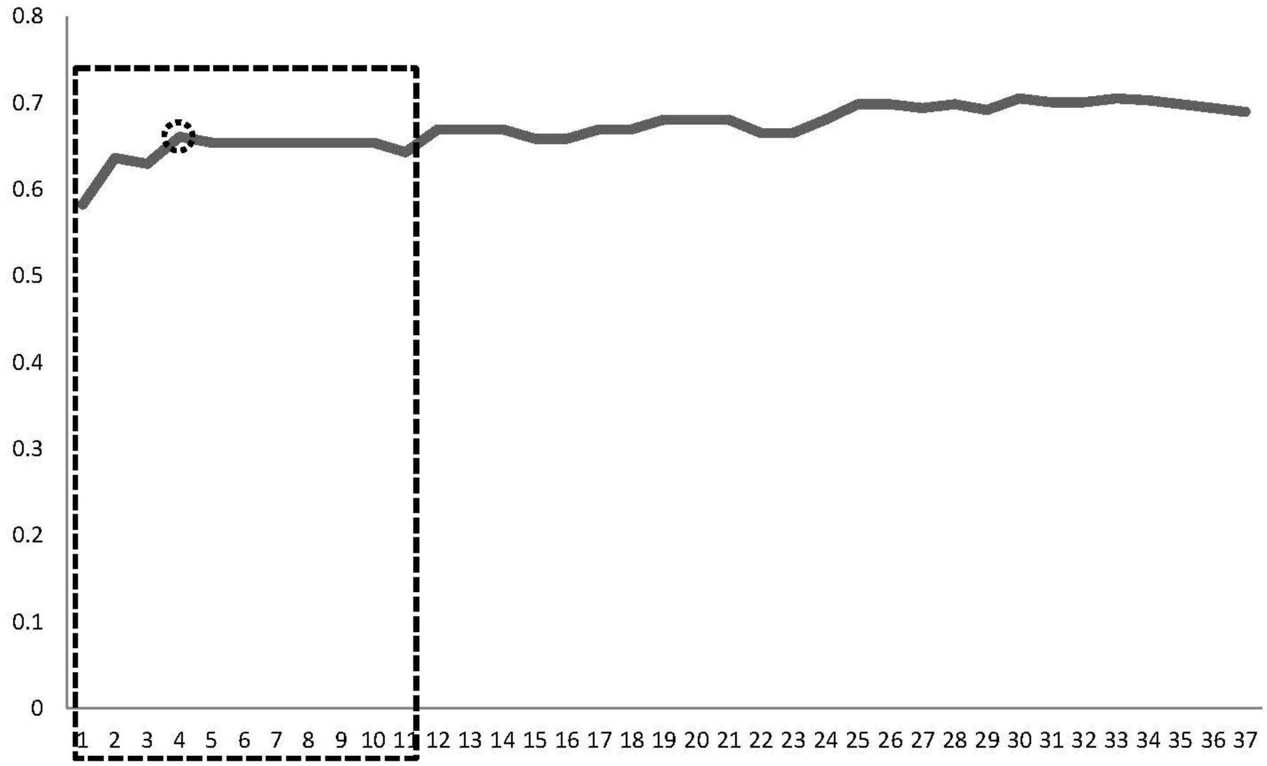

Supplementary Figure S4 Trajectory of the prediction power of the pivotal gene panel when one gene is added at one time. The GSE24450 dataset and 10-fold cross validation (with SVM as the kernel) were used.

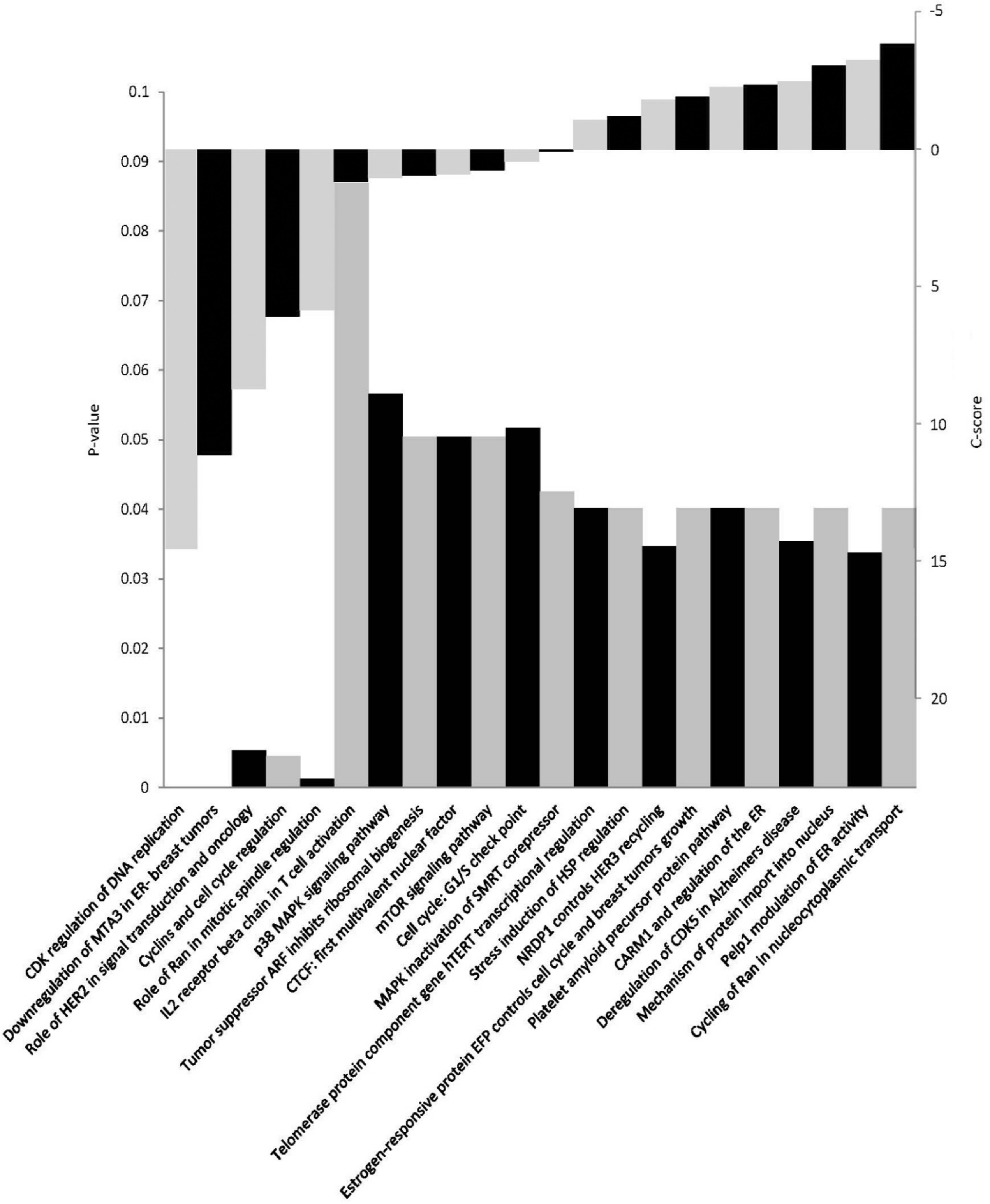

Supplementary Figure S5. The C-scores and p-values of enriched pathways for genes in the integrated diagnostic network.

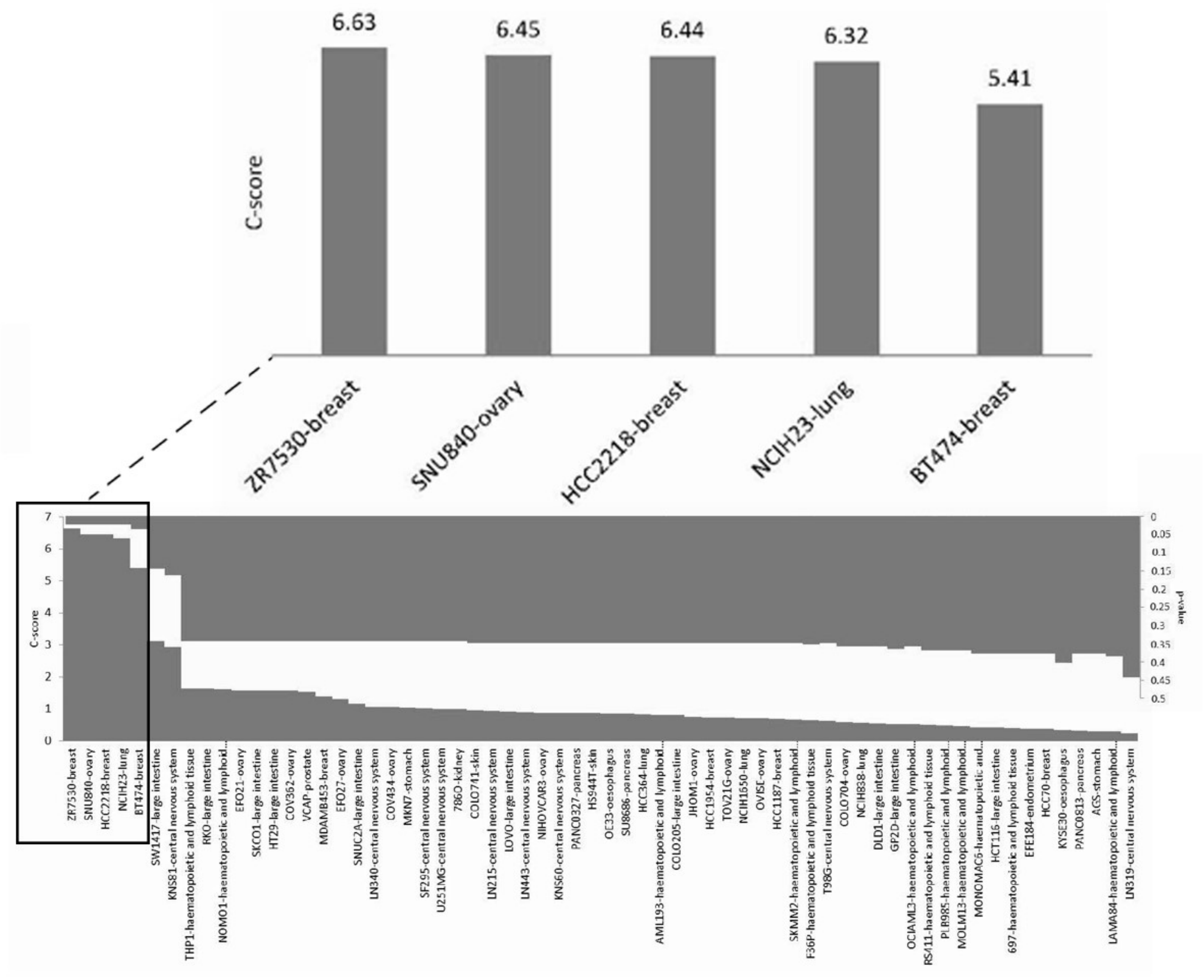

Supplementary Figure S6. The C-score and p-value of each enriched cell line from the cancer proliferation marker enrichment analysis.

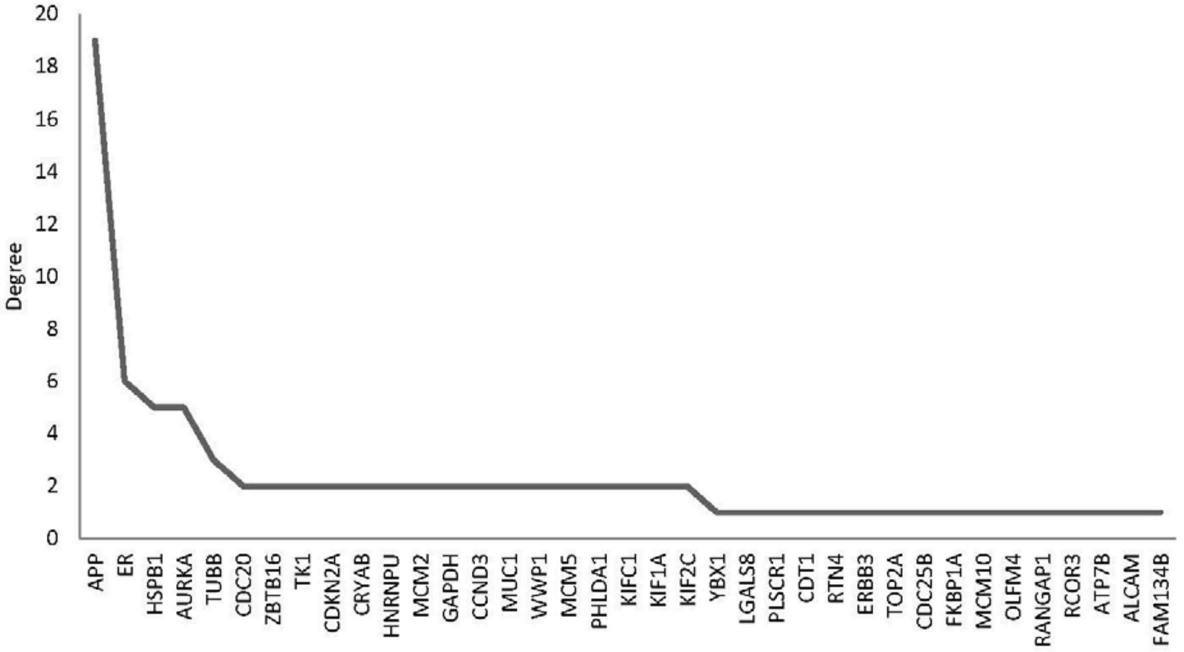

Supplementary Figure S7. Connectivity of genes in the integrated diagnostic network in BioGRID.

Supplementary Tables

Supplementary Table S1. Diagnostic gene networks constructed by merging each of the six gene expression data sets with the protein interaction network retrieved from BioGRID. ‘Node’ and ‘Edge’ each refers to the number of genes/proteins and interactions, respectively. Each column shows one diagnostic diff-gene network, which is named by concatenating the gene expression dataset with 'PPI' (representing BioGRID) by ‘&’.

	GSE70947&PPI	GSE18229&PPI	GSE15852&PPI	GSE20711&PPI	GSE65194&PPI	GSE65212&PPI
Node	50	45	47	49	41	54
Edge	49	60	54	55	44	53

Supplementary Table S2. Degree enrichment of genes in the integrated diagnostic network. 'Degree_IDN' and 'Degree_BioGRID' each shows the degree of a given gene in the integrated diagnostic network and BioGRID protein interaction network, respectively, and the 'Enrichment' is computed as the percentage of 'Degree_IDN' in 'Degree_BioGRID'.

Genes	Degree_IDN	Degree_BioGRID	Enrichment	Genes	Degree_IDN	Degree_BioGRID	Enrichment
FAM134B	1	3	33.33%	TOP2A	1	42	2.38%
KIF2C	2	7	28.57%	MCM2	2	94	2.13%
ALCAM	1	4	25.00%	HER3	1	52	1.92%
KIF1A	2	8	25.00%	HNRNPU	2	108	1.85%
KIFC1	2	11	18.18%	CRYAB	2	111	1.80%
PHLDA1	2	13	15.38%	CDKN2A	2	112	1.79%
ATP7B	1	15	6.67%	HSPB1	5	286	1.75%
RCOR3	1	16	6.25%	TK1	2	118	1.69%

RANGAP1	1	23	4.35%
AURKA	5	117	4.27%
WWP1	2	47	4.26%
MCM5	2	47	4.26%
MUC1	2	51	3.92%
CCND3	2	52	3.85%
OLFM4	1	31	3.23%
FKBP1A	1	34	2.94%
MCM10	1	34	2.94%
CDC25B	1	38	2.63%
GAPDH	2	76	2.63%
RTN4	1	60	1.67%
CDT1	1	62	1.61%
ZBTB16	2	127	1.57%
PLSCR1	1	76	1.32%
CDC20	2	154	1.30%
ER	6	571	1.05%
LGALS8	1	98	1.02%
YBX1	1	103	0.97%
TUBB	3	310	0.97%
APP	19	2346	0.81%

Supplementary Table S3. Statistics of pathways enriched by genes present in the integrated diagnostic network. Genes from the integrated diagnostic network and enriched in a given pathway are listed accordingly as ‘Genes’.

Pathways	p-value	Z-score	C-score	Genes
CDK regulation of DNA replication	7.15E-05	-1.5235	14.54	CDT1;MCM5;MCM2
Downregulation of MTA3 in ER^{-} breast tumors	0.0000715	-1.1643	11.11	HSPB1;ER;GAPDH
Role of HER2 in signal transduction and oncology	0.005373	-1.6685	8.72	HER3;ER
Cyclins and cell cycle regulation	0.004522	-1.1252	6.07	CCND3;CDKN2A
Role of Ran in mitotic spindle regulation	0.001329	-0.8836	5.85	RANGAP1;AURKA
IL-2 receptor beta chain in T cell Activation	0.086839	-0.472	1.15	CCND3
p38 MAPK signaling pathway	0.05665	-0.354	1.02	HSPB1
Tumor suppressor ARF inhibits ribosomal biogenesis	0.05036	-0.3146	0.94	CDKN2A
CTCF: first multivalent nuclear factor	0.05036	-0.2945	0.88	CDKN2A
mTOR signaling pathway	0.05036	-0.2468	0.74	FKBP1A
Cell cycle: G1/S check point	0.05174	-0.1397	0.41	CDKN2A
MAPK inactivation of SMRT corepressor	0.04262	-0.0154	0.05	ZBTB16
Overview of telomerase protein component gene hTERT transcriptional regulation	0.04019	0.3392	-1.09	ER
Stress induction of HSP regulation	0.04019	0.3822	-1.23	HSPB1
Neuroregulin receptor degredation protein-1 controls HER3 receptor recycling	0.03473	0.5433	-1.83	HER3
Estrogen-responsive protein EFP controls cell cycle and breast tumors growth	0.04019	0.6043	-1.94	ESR1
Platelet amyloid precursor protein pathway	0.04019	0.7068	-2.27	APP
Mechanism of protein import into the nucleus	0.04019	0.7225	-2.32	RANGAP1
Deregulation of CDK5 in Alzheimers disease	0.03542	0.7419	-2.48	APP
CARM1 and regulation of ER	0.04019	0.9624	-3.09	ER

PELP1 modulation of ER activity	0.03379	0.9637	-3.26	ER
Cycling of Ran in nucleocytoplasmic transport	0.04019	1.2025	-3.86	RANGAP1

Supplementary Table S4. Enriched GO terms with adjusted p-values <0.01 for genes in the integrated diagnostic network.

Biological processes	Adjusted p-value	Z-score	C-score	Genes
mitotic cell cycle (GO:0000278)	4.85E-09	-2.30482	44.12269	TOP2A;CDC20;CDT1;CDKN2A;TUBB;MCM10;KIF2C;MCM5; RANGAP1;AURKA;MCM2;CDC25B
mitotic cell cycle phase transition (GO:0044772)	1.27E-05	-2.32311	26.18521	CDT1;CDKN2A;TUBB;MCM10;MCM5;MCM2;CDC25B; AURKA
cell cycle phase transition (GO:0044770)	1.27E-05	-2.31886	26.13733	CDT1;CDKN2A;TUBB;MCM10;MCM5;MCM2;CDC25B; AURKA
cell division (GO:0051301)	3.65E-05	-2.18487	22.3278	TOP2A;CCND3;CDKN2A;TUBB;ZBTB16;MCM5
microtubule-based process (GO:0007017)	2.27E-04	-2.42233	20.32195	APP;KIFC1;TUBB;KIF2C;KIF1A;GAPDH;CRYAB;AURKA
regulation of cell cycle process (GO:0010564)	3.87E-04	-2.44626	19.21739	TOP2A;CDC20;APP;CDT1;MUC1;CDKN2A;CDC25B;AURKA
regulation of mitotic cell cycle (GO:0007346)	8.45E-04	-2.44635	17.3103	TOP2A;CDC20;APP;MUC1;CDKN2A;CDC25B;AURKA
microtubule cytoskeleton organization (GO:0000226)	8.09E-04	-2.38096	16.9527	KIFC1;TUBB;KIF2C;GAPDH;CRYAB;AURKA
cell cycle G1/S phase transition (GO:0044843)	8.95E-04	-2.21786	15.56549	CDT1;CDKN2A;MCM10;MCM5;MCM2
G1/S transition of mitotic cell cycle (GO:0000082)	8.95E-04	-2.21565	15.54995	CDT1;CDKN2A;MCM10;MCM5;MCM2
positive regulation of cell cycle (GO:0045787)	1.06E-03	-2.49653	17.10273	APP;MUC1;CCND3;CDKN2A;CDC25B;AURKA
posttranscriptional regulation of gene expression (GO:0010608)	1.18E-03	-2.44104	16.46076	APP;CDKN2A;HSPB1;HNRNPU;YBX1;GAPDH;AURKA
neuron apoptotic process (GO:0051402)	3.57E-03	-2.44341	13.76805	APP;HER3;GAPDH
positive regulation of cell cycle process (GO:0090068)	3.57E-03	-2.41793	13.62449	APP;MUC1;CDKN2A;CDC25B;AURKA
spindle assembly (GO:0051225)	3.57E-03	-2.30274	12.97543	KIFC1;TUBB;AURKA
regulation of mitotic cell cycle phase transition (GO:1901990)	5.82E-03	-2.35978	12.14544	TOP2A;CDC20;APP;MUC1;CDKN2A
neuron death (GO:0070997)	5.82E-03	-2.30657	11.87155	APP;HER3;GAPDH

regulation of cell cycle phase transition (GO:1901987)	6.96E-03	-2.34442	11.64452	TOP2A;CDC20;APP;MUC1;CDKN2A
DNA unwinding involved in DNA replication (GO:0006268)	7.82E-03	-2.1637	10.49719	TOP2A;MCM2
DNA duplex unwinding (GO:0032508)	7.82E-03	-2.12354	10.30237	TOP2A;MCM5;MCM2
DNA geometric change (GO:0032392)	7.98E-03	-2.13732	10.32432	TOP2A;MCM5;MCM2
**Molecular function**				
microtubule binding (GO:0008017)	2.57E-03	-2.41618	14.40624	KIFC1;KIF2C;KIF1A;GAPDH;CRYAB
tubulin binding (GO:0015631)	4.42E-03	-2.40501	13.03893	KIFC1;KIF2C;KIF1A;CRYAB;GAPDH
ATP binding (GO:0005524)	4.42E-03	-2.38719	12.94234	TOP2A;ATP7B;HER3;KIFC1;HNRNPU;MCM5;KIF2C;TK1;KIF1A;MCM2;AURKA
protein kinase binding (GO:0019901)	5.88E-03	-2.51336	12.9074	TOP2A;CCND3;CDKN2A;HSPB1;AURKA;CDC25B
kinase binding (GO:0019900)	8.46E-03	-2.51344	11.9942	TOP2A;CCND3;CDKN2A;HSPB1;CDC25B;AURKA
**Cellular component**				
nucleoplasm (GO:0005654)	1.12E-04	-2.24004	20.3722	TOP2A;CDC20;CDT1;CDKN2A;HNRNPU;MCM10;MCM5;YBX1;ER;MCM2;CDC25B
cytosol (GO:0005829)	8.33E-05	-2.0972	19.69824	APP;CDT1;CDKN2A;TUBB;ZBTB16;WWP1;HSPB1;RANGAP1;CDC25B;AURKA;FKBP1A;CDC20;PLSCR1;KIF2C;TK1;GAPDH;CRYAB
microtubule cytoskeleton (GO:0015630)	1.12E-04	-2.10214	19.11799	KIF2C;GAPDH;CRYAB;AURKA;MCM2
perinuclear region of cytoplasm (GO:0048471)	2.69E-03	-2.32234	13.74392	CDC20;APP;ATP7B;OLFM4;RANGAP1;AURKA
kinesin complex (GO:0005871)	2.69E-03	-2.18677	12.94157	KIFC1;KIF2C;KIF1A
extracellular vesicular exosome (GO:0070062)	3.14E-03	-2.10921	12.15975	APP;TUBB;WWP1;HSPB1;YBX1;OLFM4;RTN4;FKBP1A;MUC1;PLSCR1;ALCAM;LGALS8;GAPDH;CRYAB
nuclear envelope lumen (GO:0005641)	2.69E-03	-1.57019	9.292615	APP;TUBB
MCM complex (GO:0042555)	3.14E-03	-1.54928	8.931689	MCM5;MCM2
microtubule (GO:0005874)	6.98E-03	-2.26233	11.23045	KIFC1;TUBB;KIF2C;KIF1A;AURKA
Z disc (GO:0030018)	6.99E-03	-1.99822	9.916492	FKBP1A;HSPB1;CRYAB
